# Bridging the gaps in innovation research: A bibliometric analysis and multi-level framework integrating meso-perspectives and well-being

**DOI:** 10.1371/journal.pone.0356731

**Published:** 2026-08-24

**Authors:** Yousif Elsamani, Cristian Mejia, Yuya Kajikawa

**Affiliations:** 1 Institute for Future Initiatives, The University of Tokyo, Tokyo, Japan; 2 Department of Innovation Science, School of Environment & Society, Institute of Science Tokyo, Tokyo, Japan; Bogazici University, TÜRKIYE

## Abstract

Innovation and innovativeness frameworks are crucial for guiding progress, yet often remain fragmented, failing to connect individual behaviors, organizational ecosystems, and systemic goals, and frequently overlooking well-being. This study addresses these limitations through a large-scale bibliometric analysis of 49,398 innovation and 1,346 innovativeness publications. We employed a mixed-methods approach, combining citation network clustering with a qualitative content analysis of seminal works to map the field’s evolutionary trajectory. Our analysis confirms a structural divide: innovation frameworks typically adopt a macro-level perspective (e.g., systems, policy), while innovativeness frameworks focus on the micro-level (e.g., individual adoption). Critically, the meso-level—essential for bridging micro-actions and macro-outcomes—remains underdeveloped, and well-being is marginalized. To bridge these gaps, we propose the Integrated Framework for Innovation and Well-being (IFIW). Grounded in a multi-level architecture (Micro, Meso, Macro) and eight guiding principles, the IFIW leverages meso-level mechanisms for scalability and explicitly embeds well-being, ethics, and sustainability as core priorities. This research contributes an empirically grounded taxonomy of evolving research streams and a unified model to guide the development of innovation ecosystems that are effective, equitable, and aligned with long-term well-being.

## 1. Introduction

Innovation is a pivotal driver of technological advancement, economic growth, and societal well-being [1,2]. It enables organizations to design strategies that enhance competitiveness, adaptability, and systemic resilience [2]. However, despite their transformative potential, existing frameworks on innovation remain fragmented, often isolating individual, organizational, and systemic perspectives rather than integrating them into a cohesive model [1,3]. This siloed approach limits our understanding of how innovation dynamics interact across micro (individual/firm), meso (organizational/network/industry), and macro (systemic/societal) levels, hindering the development of equitable and sustainable solutions [3].

Traditional innovation research prioritizes technological efficiency and economic metrics, frequently overlooking ethical considerations, environmental sustainability, and human well-being [4–6]. For instance, while AI-driven governance models demonstrate potential to enhance public value, their design often neglects participatory mechanisms that align innovation outcomes with societal needs [7]. Similarly, regional innovation systems in developing economies face structural barriers due to insufficient integration of micro-level entrepreneurial capabilities with macro-level institutional support [8]. Such disconnects underscore the urgency for frameworks that reconcile multi-level interactions while embedding holistic metrics of success.

A critical gap persists in the explicit integration of well-being—a cornerstone of sustainable development—into innovation frameworks [9]. Contemporary research emphasizes that societal progress increasingly depends on non-economic factors such as social cohesion, job satisfaction, and quality of life [10]. Yet, these dimensions are rarely operationalized in innovation assessments. For example, while subjective well-being (SWB) is recognized as a vital indicator of social innovation impact, few frameworks systematically incorporate it alongside traditional performance metrics [11]. This omission persists despite global mandates like the UN Sustainable Development Goals (SDGs), which advocate for innovation ecosystems that harmonize economic, environmental, and social priorities [12,13].

The urgency for integrated innovation frameworks becomes particularly evident when, for example, examining contemporary challenges in sustainability and digital transformation. Recent studies reveal how the micro-macro disconnect creates practical barriers to addressing complex societal issues. For instance, research on open eco-innovation and Industry 5.0 documents persistent tensions between firm-level economic priorities and macro-level environmental imperatives, with companies struggling to align short-term profitability with long-term sustainability goals [14,15]. These studies consistently point to meso-level networks—such as industry consortiums and collaborative platforms—as critical but underutilized mechanisms for bridging this divide.

Similarly, the rapid deployment of AI-enabled governance systems highlights the consequences of inadequate multi-level integration. When innovation frameworks fail to connect individual user experiences with systemic policy goals, ethical risks such as algorithmic bias emerge, potentially undermining societal trust [7,16]. These challenges underscore how fragmented approaches to innovation can exacerbate rather than solve complex social problems.

Despite growing awareness of these multi-level tensions, comprehensive reviews that systematically map innovation frameworks’ thematic evolution across analytical levels remain scarce. This gap hinders the development of integrated approaches needed to address today’s interconnected challenges and foster truly resilient innovation ecosystems.

This study aims to develop a multi-level framework for innovativeness and innovation that addresses the disconnect between different analytical scales and incorporates context-sensitive considerations beyond technological efficiency and economic metrics. To achieve this, we conducted a comprehensive bibliometric analysis of 49,398 publications on innovation and 1,346 on innovativeness. Through citation network clustering and semantic similarity analysis, we identified dominant thematic structures and assessed their distribution across the micro, meso, and macro levels. Based on these insights, we explore the underdeveloped role of meso-level mechanisms as bridges between individual-level drivers and systemic outcomes, with particular emphasis on integrating well-being into innovation frameworks. Drawing from our analysis, we propose the Integrated Framework for Innovation and Well-being (IFIW), which is grounded in eight guiding principles emphasizing interconnectedness, scalability, ethical integrity, and long-term societal relevance. This study makes three key contributions: (1) a taxonomy of existing innovation frameworks, (2) evidence-based insights into structural gaps across levels, and (3) a unified model that aligns innovation outcomes with sustainable development and societal well-being. By linking individual innovativeness, organizational agility, and systemic integration, the IFIW advances academic discourse and provides actionable guidance for designing inclusive and adaptive innovation ecosystems.

## 2. Literature Review

### 2.1. Innovation and Innovativeness Frameworks: Definitions, Focus, and Applications

Innovation frameworks serve as structured conceptual tools that define, guide, and facilitate innovation processes across different contexts, including organizational, systemic, and ecosystem levels [17,18]. These frameworks help organizations and policymakers design and implement innovation strategies effectively. In contrast, innovativeness frameworks focus on the capacity of individuals and organizations to adopt, implement, and sustain innovations, emphasizing factors such as absorptive capacity, organizational culture, and leadership [19–21].

The application of these frameworks spans multiple domains, including healthcare, technology, and organizational development. Hernandez et al. proposed a patient-centered innovation framework in healthcare, underscoring the importance of leadership, strategic alignment, and feedback mechanisms in fostering innovation [17]. In the technological domain, Narvekar and Jain introduced a cognitive framework for technological innovation, which aids in organizational restructuring and competence building [18]. Woolthuis et al. contributed to the policy perspective by developing a system failure framework, which focuses on overcoming structural barriers in innovation systems through targeted policy interventions [22]. Granstrand and Holgersson further refined the concept of innovation ecosystems, defining them based on the interactions of key actors, activities, and institutional environments [23]. Additionally, Quintane et al. advanced a knowledge-based framework, emphasizing the role of knowledge flows and their outcome-oriented nature in shaping innovation processes [24].

### 2.2. Complexity in Innovation Frameworks and Levels of Analysis

Innovation frameworks exhibit inherent complexity due to their engagement with multiple stakeholders, interdependencies, and the operationalization of knowledge [25]. The analysis of these frameworks occurs at three primary levels: micro (individuals or firms), meso (networks and industries), and macro (regulatory and societal systems), each introducing unique challenges and considerations [26]. The need to navigate these multilevel dynamics is particularly pronounced in sustainability-driven innovations, where tensions between economic and environmental goals influence value creation and capture at different levels [14].

Researchers have proposed diverse frameworks tailored to specific innovation challenges to address this complexity. Krause and Schutte developed an open innovation framework incorporating continuous improvement cycles, benefiting small and medium-sized enterprises (SMEs) by enhancing their adaptability and knowledge integration [27]. Such frameworks resonate with findings from the maritime industry, where firms participating in open eco-innovation networks demonstrated a cautious approach, often prioritizing economic sustainability at the micro and meso levels while struggling to commit resources toward macro-level environmental goals [14]. Mostafavi et al. emphasized the necessity of a system-of-systems approach to resolving methodological challenges in innovation studies, advocating for a more interconnected and holistic perspective [28]. This approach aligns with geofencing technology studies, highlighting how institutional-level barriers and the need for public-private collaboration influence innovation uptake across micro, meso, and macro levels [29].

At multiple levels of innovation analysis, Anderson et al. proposed a comprehensive framework for creativity and innovation, examining how innovation unfolds at individual, team, and organizational levels [30]. Their approach echoes findings from sustainable transport start-ups, which require legitimacy-building efforts across meso (industry networks) and macro (regulatory) levels to integrate electric and autonomous vehicles into transport ecosystems successfully [31]. Aslam et al. responded to contemporary industrial shifts by introducing the Absolute Innovation Management framework, which aligns innovation processes with the principles of Industry 5.0, focusing on human-centered and sustainable innovation [15]. Similarly, the micro-meso-macro assessment of geofencing implementation reveals that achieving sustainability goals necessitates addressing regulatory constraints, aligning business models, and fostering technological adoption through cross-level interactions [29].

Rowley et al. further addressed the complexity of innovation typologies by developing an innovation-type mapping tool, offering a structured means of categorizing and integrating different forms of innovation [32]. The importance of mapping innovation categories is evident in the study of sustainable freight transport, where start-ups engage in meso and macro-level networking activities to gain legitimacy and integrate their solutions into broader industrial ecosystems [31]. These findings underscore the necessity of an analytical framework that accommodates interactions across multiple levels to understand how innovations evolve, diffuse, and encounter barriers in different contexts. By incorporating these multilevel perspectives, this study builds on existing innovation frameworks while addressing the systemic interdependencies that shape innovation outcomes in real-world applications.

### 2.3. Bibliometric Studies on Innovation and Innovativeness Frameworks

In addition to the above qualitative and case-based analysis, bibliometric analyses provide valuable insights into the research trends, thematic focus, and evolution of concepts within innovation studies [33–35]. Such analyses help identify knowledge gaps, emerging research areas, and theoretical underpinnings that shape the field. Despite the significance of innovation and innovativeness frameworks, a targeted bibliometric study explicitly focusing on these frameworks has not been identified. However, several bibliometric studies have explored related innovation concepts. Quaiser and Pandey analyzed the role of design thinking in innovation, highlighting its cross-industry applications and effectiveness in problem-solving [34]. Schmitz et al. examined innovation and entrepreneurship trends in academic research, identifying a fragmented and under-theorized body of literature [36]. Wisdom et al. conducted a bibliometric review of adoption frameworks, emphasizing key factors that influence the adoption and implementation of innovation [37]. Chew applied bibliometric methods to develop an integrative design framework for service innovation, advocating for customer-centric innovation models [38].

Additionally, Anderson et al. reviewed bibliometric trends in creativity and innovation, focusing on integrating innovation across multiple levels [30]. Other bibliometric-based studies have looked at employee innovativeness and its connection to well-being presenting a holistic framework of the relationship between them and advocating for interconnected and multilevel approach for evaluating or enhancing these constructs [35]. These studies illustrate the diverse research trajectories within innovation scholarship while underscoring the need for systemic and multi-level analysis of innovation frameworks—a gap this study addresses.

### 2.4. Development Principles of Innovation Frameworks and the Role of Well-Being

The development of innovation frameworks relies on foundational principles that ensure adaptability, effectiveness, and alignment with organizational goals. Common principles include iterative development, stakeholder integration, and systematic problem resolution [39–42]. Several scholars have contributed to refining these principles. Kavadias and Hutchison-Krupat proposed a conceptual framework for managing innovation, focusing on the key phases of ideation, selection, and execution [39]. Adamides and Karacapilidis integrated knowledge and social dynamics into innovation frameworks, presenting a systemic methodology for problem resolution [40]. Lobo and Samaranayake introduced an innovation management assessment framework, combining Lean Six Sigma principles with stage-gate models to improve innovation decision-making [43]. Patiniotakis et al. developed the Unified Collaborative Innovation Framework (UCIF), incorporating participatory innovation approaches and knowledge management strategies [41]. Additionally, Shrivastava and Souder proposed a strategic management model for new product development, identifying critical success variables and research hypotheses to optimize the innovation process [42]. Despite the extensive research on innovation frameworks, well-being has not been a central focus in innovation research. However, its growing importance in human development theory and sustainability discourse suggests that well-being should be integrated into innovation frameworks to ensure that innovation delivers long-term, equitable societal benefits.

The literature on innovation and innovativeness frameworks demonstrates the field’s complexity, multi-level dynamics, and evolving theoretical foundations. While various frameworks have been developed to address sectoral and organizational needs, critical gaps remain. First, a comprehensive overview of the field taxonomy and the evolution of its research topics while evaluating their distribution across micro, meso, and macro levels is lacking. Second, well-being is rarely operationalized in innovation research despite its centrality to sustainable progress. Third, holistic frameworks and principles to inform the future development of innovation frameworks across all levels are lacking. Addressing these gaps is central to this study’s contribution.

## 3. Data and methods

### 3.1. Data

The bibliographic data utilized in this research was obtained from the Web of Science (WoS) Core Collection, a comprehensive database spanning multiple disciplines and document categories. This includes the Science Citation Index Expanded, Social Sciences Citation Index, Arts & Humanities Citation Index, Emerging Sources Citation Index, Book Citation Index, and Conference Proceedings Citation Index. Previous studies have demonstrated that WoS provides journal article coverage comparable to that of Scopus [44], further solidifying its utility in scholarly research. Moreover, WoS data is widely recognized for its accessibility in bibliometric analyses and its reliability for citation network mapping and data-driven research applications [45]. Its structured format and extensive metadata make it a preferred resource for large-scale scientometric investigations.

To identify studies related to innovation frameworks, the topical search query TS = “innovation” AND “framework*” was employed, while the query TS = “innovativeness” AND “framework*” was used for innovativeness frameworks. The asterisk (*) functioned as a truncation symbol to capture variations of the terms, such as “frameworks” and “frameworking.” This specific combination of search terms was chosen to ensure the retrieval of literature that explicitly proposes, applies, or critiques conceptual structures (frameworks), rather than the vast body of literature that merely mentions innovation or innovativeness as a general variable. These topical searches retrieved records containing the specified terms within the title, abstract, or keywords. No temporal restrictions were applied, allowing for the inclusion of all records available in the database. Data collection was completed on October 10, 2024, yielding 49,398 records for innovation frameworks and 1,346 for innovativeness frameworks across all document types.

### 3.2. Methods

This study employed three distinct methodologies to analyze topical trends, examine semantic similarities, and investigate focus levels. Our approach builds upon established analytical frameworks in scientometrics that successfully utilize advanced text-mining and clustering techniques to map complex interdisciplinary fields. For instance, recent studies have demonstrated the efficacy of hierarchical clustering and multidimensional scaling for visualizing knowledge evolution [46], as well as the utility of text classification for filtering and contextualizing large-scale health innovation datasets [47]. Following these methodological precedents, the first method involves identifying topics by clustering a direct citation network of publications. A direct citation network links two academic articles when one cites the other, a technique shown to reveal research field taxonomies [48,49] and research fronts [50]. This approach is particularly effective when analyzing long-term datasets, as in this study. While alternative network types like co-citation [51] and bibliographic coupling [52] networks exist, they are better suited for different objectives and time frames. In constructing the citation network, each article serves as a node, and links represent citations between articles. Groups of nodes with denser intra-cluster connections compared to inter-cluster connections form clusters. Optimal partitioning of the network is achieved by maximizing modularity, a measure of community structure strength [53].

To identify distinct research topics within each domain, this study applied the Louvain community detection algorithm implemented in the Python-igraph library (version 0.11.3), using a resolution parameter of γ = 1.0 to divide each citation network into clusters; this method was selected for its computational efficiency in handling large networks [54]. Once clusters were identified, summary statistics such as publication years and citation counts were computed. Clusters were named based on a triangulation of three data points: (1) an algorithmic extraction of the most frequent keywords within the cluster and relevant metadata (e.g., journal names, countries, or authors), (2) a qualitative content review of the top 20 most cited articles within each cluster, using citation impact as a proxy for thematic influence and representativeness, and (3) an examination of the cluster’s citation relationship to other groups —specifically analyzing inter-cluster links to determine the cluster’s structural role within the network. This multi-faceted approach ensured that cluster labels reflected both the semantic content and the structural role of the research theme. The same triangulation logic also informed our higher-order interpretation of thematic streams, consistent with bibliometric triangulation approaches that combine multiple analytical perspectives to strengthen field-level interpretation [55].

The second method assessed semantic similarity between innovation and innovativeness clusters and subclusters using cosine similarity. This analysis was implemented with the sentence-transformers library using the pre-trained all-MiniLM-L6-v2 model, a BERT-based model that produces 384-dimensional dense vector embeddings and is optimized for high-quality semantic representation with strong computational efficiency [56]. Unlike bag-of-words approaches, BERT analyzes text bidirectionally and interprets words in context, enabling it to capture nuanced meanings, semantic relationships, and domain-specific vocabulary more effectively [57,58]. To construct thematic profiles, we aggregated the titles and abstracts of all publications within each innovation subcluster and innovativeness cluster into a single text representation. Cosine similarity scores were then computed between innovation subclusters and innovativeness clusters, with values closer to 1 indicating stronger semantic similarity and values closer to 0 indicating weaker similarity. To visualize cross-domain semantic proximity, we created a similarity-profile matrix from these pairwise scores and set within-dataset similarities to zero to highlight relationships between the two domains. The resulting profile vectors were then projected into two dimensions using UMAP with n_components = 2, metric = “cosine,” min_dist = 0.25, and random_state = 100 for reproducibility.

The third method, unique to this study, evaluated the focus level of each topic. A term dictionary was developed to classify terms associated with micro, meso, and macro focus levels. To systematically assess the thematic focus of research articles, a structured term dictionary was developed, categorizing terms based on their alignment with micro, meso, and macro levels of analysis. This approach builds upon a micro-meso-macro framework that has been widely applied in innovation and sustainability studies to capture the interplay of firm-level activities, network-level interactions, and systemic societal influences. The dictionary was constructed through an iterative process, incorporating insights from three key studies that explicitly address the multilevel nature of innovation and sustainability transitions. The first study, focusing on open eco-innovation in the maritime industry, underscores the tension between economic and environmental value creation within multi-stakeholder networks. It highlights how firms’ conservative innovation strategies at the micro level impede broader value capture at the macro level [14]. The second study, examining start-up collaborations in sustainable freight transport, reveals the critical role of networking activities at the meso level in legitimizing innovations within transport ecosystems [29]. Finally, the third study on geofencing for sustainable transport provides a structured analysis of drivers and barriers across institutional levels, emphasizing the need for cross-level collaboration to overcome implementation challenges [31]. To ensure the validity and precision of these categories, the dictionary underwent a manual verification process. A random sample of 50 abstracts containing the identified terms was reviewed to confirm that the terms (e.g., ‘ecosystem,’ ‘ technology adoption’) were consistently used in the context of the intended level of analysis, and ambiguous terms were removed to minimize false positives.

Drawing from these studies, distinct terminology associated with each level was identified and compiled into a term dictionary (Table 1). Micro-level terms capture firm-centric activities, entrepreneurial strategies, and direct innovation processes. Meso-level terms emphasize network dynamics, industry collaborations, and inter-organizational linkages. Macro-level terms reflect regulatory, societal, and environmental factors that shape the broader innovation landscape. Generic terms such as “firm” and “organization” were excluded from the micro-level category, as they are broadly used across all levels of analysis and could introduce ambiguity in classification. This term dictionary serves as the foundation for the quantitative assessment of topic focus, enabling a structured evaluation of how innovation discourse is distributed across different analytical levels.

**Table 1:** Focus level term dictionary.

Levels	Terms
Micro	individual, user, personal, personality, buyer, customer, consumer, start-up, product development, prototyping, technology adoption
Meso	partnership, collaboration, ecosystem, networks, network, integration, standardization
Macro	society, social, international, regional, government, legislation, regulation, policy, Infrastructure, sustainability, economic development, global supply chain

For each cluster i, the number of articles containing these terms in their titles, abstracts, or author keywords was counted: $N_{mi,i}$ for micro, $N_{me,i}\;$for meso, and $N_{ma,i}\;$for macro. The weight of each focus level within a cluster, denoted as $W_{mi,i}$, $W_{me,i}$, and $W_{ma,i}$, was calculated using equation (1):


$$\begin{array}{c} W_{mi,i}=\frac{N_{mi,i}}{N_{i}},\;W_{me,i}=\frac{N_{me,i}}{N_{i}},\;W_{ma,i}=\frac{N_{ma,i}}{N_{i}} \end{array}$$ (1)

**Fig 1:**
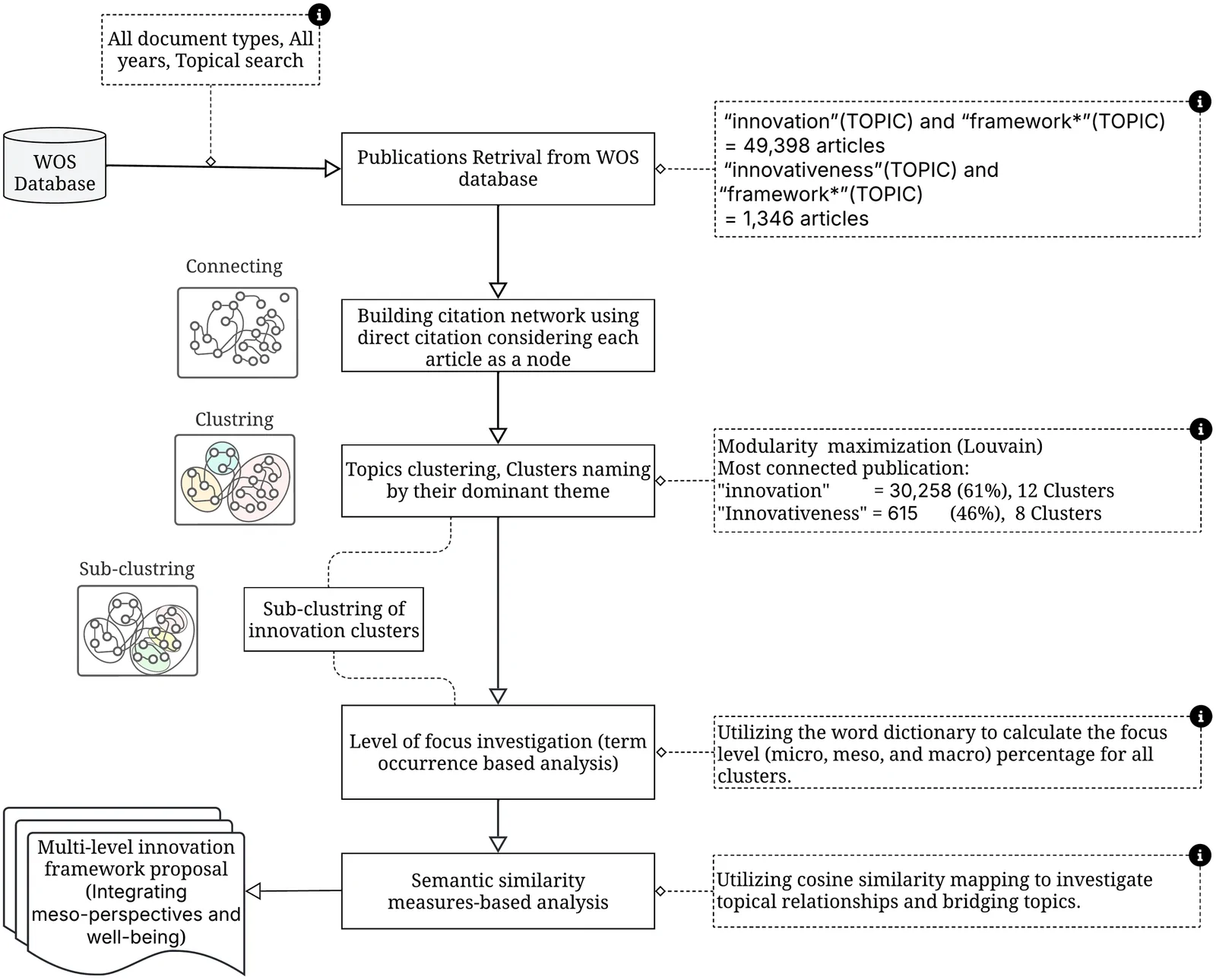
Steps and methods utilized in this study. The symbol 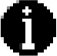 provides more information about the steps.

Where $W_{mi,i}$, $W_{me,i}$, and $W_{ma,i}$ represent the weight of the micro, meso, and macro focus levels in cluster i, respectively. $N_{mi,i}$, $N_{me,i}$, and $N_{ma,i}$ denote the number of articles in cluster i that contain terms associated with the micro, meso, and macro focus levels, respectively. $N_{i}$ is the total number of articles in cluster i. This method provides a quantitative measure of the dominant focus level for each topic. The formula is used to calculate the focus level percentage for all innovation and innovativeness clusters. The following flow chart (Fig 1) summarizes and explains the steps and methods that we followed to conduct this study.

## 4. Results

### 4.1. Focus level patterns and thematic trends

The analysis on innovation framework publications resulted in 12 main clusters formed by 30,258 connected articles. The connected articles represent about 61% of the total retrieved articles. Additionally, the analysis on innovativeness publications resulted in 8 main clusters formed by 615 connected articles. The connected articles represent about 46% of the total retrieved articles. The remaining articles in both datasets constitute ‘isolates’ or small disconnected components. These largely consist of very recent publications that have not yet accrued citations, or niche studies that do not engage with the core theoretical conversations of the field. While their exclusion is necessary to map the consolidated structural backbone of the discipline, we acknowledge that this may filter out emerging or peripheral perspectives not yet integrated into the mainstream discourse.

To investigate the general focus level for each cluster, we calculated the percentage for all three levels (micro, meso, and macro) using the approach detailed in the methods section. The tables below summarize the clustering results showing the cluster ID, cluster name, The number of articles in each cluster (N), the percentage of articles (PCT), the average publication year (APY), and the focus level percentages. The full list of innovation subclusters (with key quantitative data) is provided in the supplementary materials of this article (S1 Table in S1 File).

Publication trends and focus level analysis reveal clear differences in how innovation and innovativeness are addressed across research clusters. As shown in Table 2, the innovation framework has expanded to include systemic and sustainability-oriented topics, with clusters such as Sustainable Innovation Management (APY = 2020.1) and Sustainable Business Model Innovation (APY = 2020.3) reflecting this trend. In contrast, as detailed in Table 3, the innovativeness framework remains more focused on user-level technology adoption, exemplified by clusters like User Adoption Factors of Personal Digital Augmentation Technologies (APY = 2020.5) and Personal Innovativeness in Technology Adoption Behavior (APY = 2019.7).

**Table 2:** Innovation framework research clusters with their number of articles (N), percentage (PCT), average publication year (APY), and level percentage (micro, meso, macro).

ID	Cluster Name	N (PCT)	APY	Micro	Meso	Macro
IN 1	Organizational Innovation Drivers and Performance Outcomes	4244 (14%)	2016.7	40%	23%	45%
IN 2	Innovation Systems Analysis	3936 (13%)	2018.4	21%	28%	89%
IN 3	Sustainable Innovation Management	3135 (10%)	2020.1	26%	23%	80%
IN 4	Innovation Ecosystem Management	2913 (10%)	2019.3	33%	30%	59%
IN 5	Knowledge Management and Innovation Networks	2355 (8%)	2016.8	29%	37%	48%
IN 6	Technology Innovation Adoption Frameworks	2319 (8%)	2018.7	39%	23%	61%
IN 7	Sustainable Business Model Innovation	2041 (7%)	2020.3	35%	27%	73%
IN 8	Academic Entrepreneurship Systems	1864 (6%)	2018.2	48%	32%	72%
IN 9	Implementation Science Frameworks	1413 (5%)	2018.4	33%	30%	71%
IN 10	Innovation Implementation Frameworks	1299 (4%)	2019.1	26%	31%	82%
IN 11	Social Innovation Theory and Framework Development	1011 (3%)	2019.4	42%	31%	73%
IN 12	Others (Responsible Innovation Governance)	3728 (12%)	2018.2	27%	28%	69%

**Table 3:** Innovativeness framework research clusters with their number of articles (N), percentage (PCT), and average publication year (APY), and level percentage (micro, meso, macro).

ID	Cluster Name	N (PCT)	APY	Micro	Meso	Macro
IS 1	Organizational Innovation Capability	118 (19%)	2015.0	46%	17%	31%
IS 2	Innovation Management and Performance Outcomes	64 (10%)	2015.5	66%	17%	27%
IS 3	User Adoption Factors of Personal Digital Augmentation Technologies	62 (10%)	2020.5	95%	15%	56%
IS 4	Individual Innovativeness as a Driver of Technology Adoption	58 (9%)	2018.9	60%	16%	60%
IS 5	Personal Innovativeness in Technology Adoption Behavior	55 (9%)	2019.7	96%	11%	58%
IS 6	Technology Acceptance Models in Human-Enhancement Systems and Healthcare Technologies	47 (8%)	2018.7	94%	13%	38%
IS 7	Innovation Management and Market Adoption Strategies	46 (7%)	2016.4	43%	17%	26%
IS 8	Others (Innovation Adoption Frameworks)	165 (27%)	2017.2	65%	18%	45%

At the subcluster level (in innovation) Policy Innovation Diffusion (IN 10−8) and Urban Resilience Innovation Systems (IN 10−9) each exhibit macro-level focus scores above 98%. These clusters explore how public institutions, regulations, and governance frameworks shape innovation outcomes. For example, highly cited studies in these clusters examine how policy entrepreneurs promote innovation adoption, how urban planning integrates innovation for resilience, and how systemic change is coordinated through open government initiatives [59]. These themes reflect a growing interest in mission-oriented and policy-driven innovation, where the state plays a central role in directing innovation toward societal challenges.

On the other hand, the innovativeness framework remains strongly micro-focused. Clusters such as User Adoption Factors of Personal Digital Augmentation (IS 3) and Personal Innovativeness in Technology Adoption (IS 5) have micro-level focus scores exceeding 95%. These clusters emphasize how individual-level factors, such as perceived usefulness, behavioral intention, and personal readiness, affect the adoption of new technologies. For example, IS 3 discusses how users interact with digital health and augmentation technologies, while IS 5 investigates the psychological drivers behind technology acceptance and resistance.

Additional insights come from micro-dominant innovation subclusters like Consumer Resistance and Adoption Patterns (IN 6–6, 80% micro) and Technology Adoption Models (IN 6–8, 63% micro), which—while situated in the innovation framework—closely resembles the focus of innovativeness clusters. These subclusters explore how user attitudes, perceptions of risk, and innovation resistance influence the uptake of mobile services and digital platforms. Highly cited articles in these areas have examined consumer perceptions of mobile service value, decision-making in the adoption of retail technologies, and the influence of digital literacy on technology readiness.

Overall, the innovation framework typically looks “from the top down,” prioritizing structural change, institutional dynamics, and system-wide coordination. In contrast, the innovativeness framework works “from the bottom up,” examining personal motivation and individual responses to innovation. While both frameworks offer valuable insights, their disconnection reveals a structural gap in the literature: the absence of a coherent approach that links individual-level behavior with macro-level innovation systems. This underscores the importance of meso-level analysis, which is further explored in the following sections.

### 4.2. Evolving Discourses and Thematic Streams

While the quantitative focus level analysis highlights the structural distinction between “top-down” innovation and “bottom-up” innovativeness, a deeper qualitative review of seminal publications reveals that these clusters are not static. Instead, the field has undergone a distinct evolutionary trajectory—shifting from internal organizational capabilities to broader systemic interactions, then to individual user psychology, and finally toward purpose-driven societal outcomes. We categorize these developments into four dominant thematic streams that cut across the identified clusters.

Stream 1: The Managerial Core (Firm-Centric Focus). The foundational stream of the literature (IN 1, IS 1) is heavily rooted in the management sciences, focusing on how organizations build the internal capacity to innovate. With the highest average citation counts (Mean: 125.8), this stream establishes the “rules” of organizational performance. Qualitative analysis of key texts reveals a strong consensus that innovation is a function of culture and learning rather than just R&D spending. Seminal works like Hurley and Hult [60] and Calantone et al. [61] argue that a firm’s “learning orientation” is the critical antecedent to innovativeness, which in turn drives performance. Woodman et al. [62] expanded this by theorizing organizational creativity as a complex interaction between individuals and their social setting, while Deshpandé et al. [63] provided empirical evidence from Japanese firms showing that corporate cultures emphasizing competitiveness and entrepreneurship significantly outperform those focused on internal cohesiveness. Collectively, this stream frames innovation as a manageable, internal capability optimized for competitive advantage.

Stream 2: The Systemic Turn (Network-Centric Focus). As the field matured (approx. 2016–2018), a “systemic turn” emerged, moving the unit of analysis from the single firm to broader networks and ecosystems. Innovation Clusters IN 2, 4, and 8 exemplify this shift. This discourse argues that innovation is co-produced by a network of actors. Teece [64] bridged the gap between firm and ecosystem by introducing “dynamic capabilities,” describing how firms must sense and seize opportunities within a complex environment. Adner and Kapoor [65] further formalized this by defining ecosystems not just as loose networks, but as structural alignments where partners interact to materialize a value proposition. At the macro level, frameworks such as “Technological Innovation Systems” (TIS) [66,67] shifted the focus to the functions—such as legitimacy creation and resource mobilization—required to support emerging technologies.

Stream 3: The Digital and User-Centric Shift (Individual-Centric Focus). A distinct and more recent stream (Innovativeness Clusters 3, 4, 5; Mean Years ~2019–2020) reflects the explosion of personal digital technology. Here, the discourse pivots sharply from the “organization” to the “individual user.” The dominant theoretical lens in this stream is the Technology Acceptance Model (TAM) and its extensions. Recent empirical studies [68,69] illustrate this discourse, moving beyond simple utility to explore “consumer acceptance” in contexts like mobile payment systems, driven by factors such as social influence and habit. This stream emphasizes individual psychological traits, where constructs like “personal innovativeness” are integrated to predict how willing a user is to embrace new tools [68,70]. Unlike the managerial stream, which views people as employees within a system, this stream views them as autonomous adopters, analyzing psychological traits—such as resistance or openness—that determine the success of digital platforms and wearables.

Stream 4: Purpose-Driven Innovation (Societal-Centric Focus). The most recent and critical stream (Innovation Clusters IN 3, 11, and 12) attempts to align innovation with broader societal goals, such as sustainability and ethics. This stream represents a normative shift in the literature. Boons and Lüdeke-Freund [71] pushed the discourse forward by arguing that business models must explicitly create “social and environmental value” alongside economic value. Stilgoe et al. [72] introduced a framework for “Responsible Innovation,” urging governance mechanisms that anticipate and deliberate on the ethical implications of science and technology. Similarly, Cajaiba-Santana [73] reconceptualized social innovation not as a niche activity but as a driver of institutional change. However, our qualitative review notes a critical fragmentation: while these texts passionately argue for sustainability, they often remain distinct from the mainstream “Managerial Core,” suggesting that well-being and purpose are still treated as specialized sub-fields rather than central tenets of general innovation theory.

### 4.3. Semantic intersections between innovation and innovativeness

**Fig 2:**
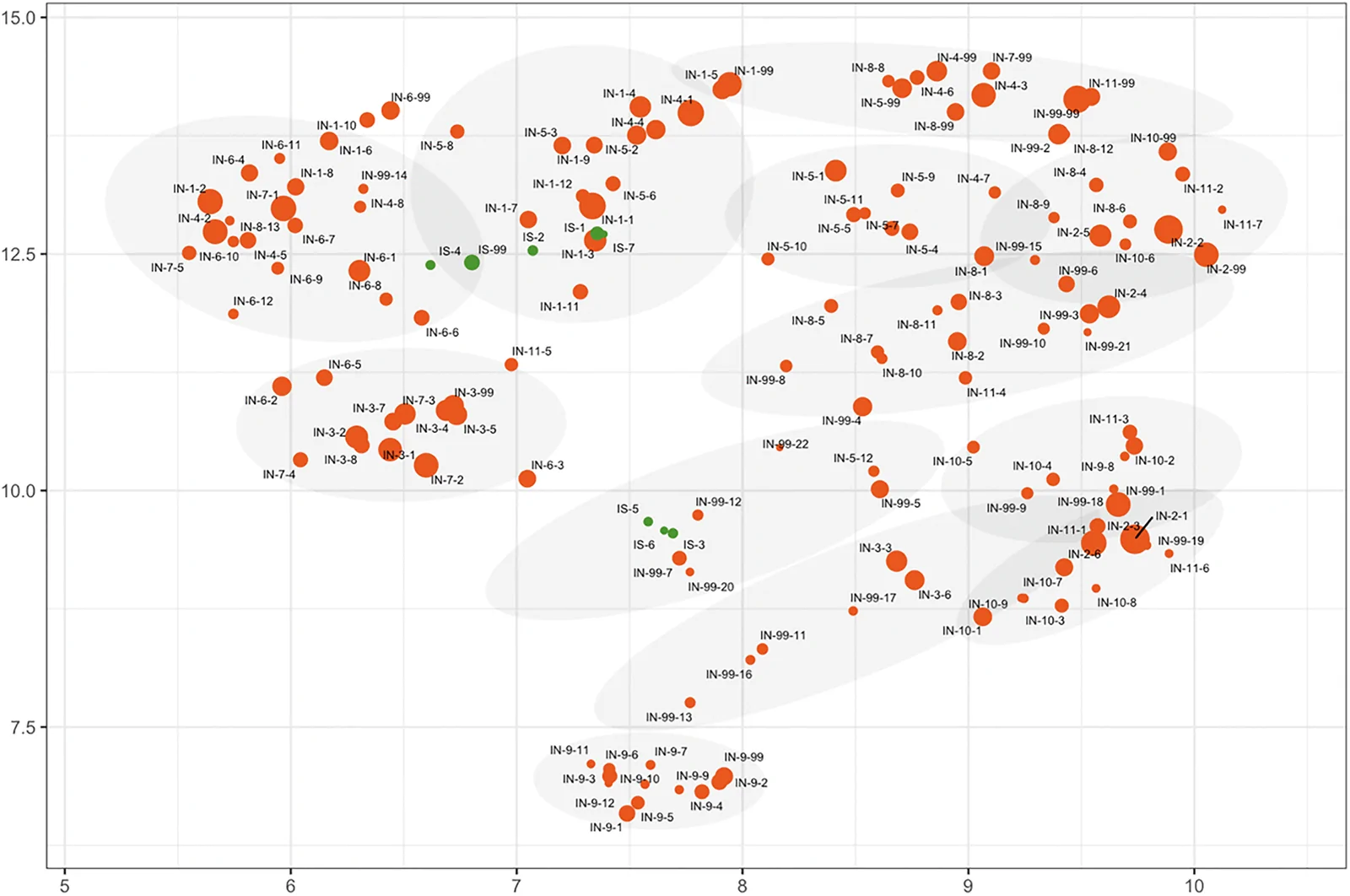
Semantic similarity map between innovativeness clusters and innovation subclusters. Each point represents an innovativeness cluster or an innovation subcluster, with color indicating affiliation (brown for innovation subclusters; green for innovativeness clusters) and size reflecting the number of documents. The x- and y-axes represent the first two UMAP projection dimensions generated from cosine-based semantic similarity profiles. These axes are dimensionless visualization coordinates and should not be interpreted as independent theoretical variables. The relative distance between points is the main information: points located closer together are more semantically similar, while more distant points are less semantically similar.

Despite the general separation between the two frameworks, semantic analysis reveals important areas of thematic overlap. These intersections highlight opportunities for conceptual integration, particularly around emerging technologies and organizational innovation. Fig 2 visualizes these relationships using UMAP to project cosine-based semantic similarity profiles into two dimensions. Because the axes are used only for visualization, their values should be interpreted as relative projection coordinates rather than substantive variables; the meaningful information is the relative proximity among clusters and subclusters.

As visualized in Fig 2, several innovativeness clusters are located near innovation subclusters in the semantic map, suggesting shared vocabulary and focus. For instance, IS 3, IS 5, and IS 6 are positioned close to innovation subclusters associated with technology adoption and digital innovation, such as IN 6–6 (Consumer Resistance and Adoption Patterns), IN 6–8 (Technology Adoption Models), and IN 6–7 (Digital Technology Adoption). These topics emphasize how users interact with advanced technologies and how their behavior influences adoption and value creation. The shared emphasis on human-centered innovation, user behavior, and cognitive drivers reveals a natural link between personal innovativeness and product- or service-level innovation. This overlap reflects a human-centric view of innovativeness and innovation, where innovations created by firms can enhance the innovativeness of individuals.

Other intersections involve clusters with broader strategic and organizational implications. Individual and organizational innovativeness in IS 1, IS 2, IS 4, and IS 7 align semantically with innovation subclusters such as Innovation-Performance Nexus (IN 1–1), Organizational Creativity (IN 1–3), and Organizational Agility (IN 5–6). These overlaps suggest a continuum from individual traits to firm-level capabilities and innovation outcomes. The literature in these clusters explores how personal innovativeness feeds into organizational processes—such as creativity, capability development, and dynamic adaptation—ultimately influencing competitiveness and market success. This overlap reflects an interactive process through which individual innovativeness can contribute to organizational innovativeness.

### 4.4. Bridging roles of meso-level subclusters

The above semantic intersections demonstrate that the gap between innovation and innovativeness frameworks is not conceptual but structural. Topics like user behavior, firm strategy, and digital transformation naturally span both domains. Recognizing and formalizing these links can help build more comprehensive frameworks that reflect the realities of multilevel innovation processes via meso-level mechanisms. Meso-level mechanisms—such as partnerships, networks, and organizational learning—play a critical role in connecting individual actions with systemic outcomes. Subclusters with high meso focus have the potential to illustrate how innovation moves across levels, enabling broader impact and scalability.

Subcluster IN 4−3 (Innovation Ecosystem Dynamics) has the highest meso-level percentage (74.5%) and focuses on how firms interact within complex ecosystems. Influential studies in this cluster describe how value is co-created through collaboration and how firms depend on the timing and alignment of their partners [74]. Another key study emphasizes the importance of ecosystem orchestration in coordinating innovation activities across multiple actors [65]. These works show that innovation does not happen in isolation—it relies on relationships, trust, and aligned incentives. Similarly, Cluster IN 5−5 (Network-Based Innovation and Knowledge Transfer) (73.8% meso) explores how organizations exchange knowledge across boundaries. Research here highlights the role of alliance portfolios, absorptive capacity, and social capital in driving innovation. One highly cited article explains how firms build strategic advantage by managing a network of partnerships, while others emphasize the importance of mutual learning and joint capability building [75]. These dynamics are essential for translating individual creativity into organizational practice and systemic transformation.

Other meso-focused clusters, such as Digital Business Transformation through Servitization (IN 4–5) and Network Innovation Management (IN 4–6), further illustrate how firms embed innovation into their operational models and strategic positioning. These clusters also engage with themes such as digital platforms, ecosystem governance, and cross-sectoral collaboration—highlighting the breadth of meso-level influence.

These findings show that the meso level is not simply an intermediate space—it is an active site of integration. It connects user behavior with policy goals, firm routines with ecosystem shifts, and short-term outcomes with long-term trajectories. Clusters at this level are especially important for designing frameworks that are both scalable and responsive to real-world complexity. Moreover, although certain groups demonstrate thematic proximity to core innovation principles—such as scalability, contextual adaptability, and cross-level feedback, these elements are inconsistently addressed and often lack structural coherence.

The bibliometric evidence converges on four core findings. First, innovation and innovativeness research remain structurally divided between macro-oriented and micro-oriented perspectives. Second, meso-level mechanisms are comparatively underdeveloped despite their pivotal role in linking localized innovation processes to broader system outcomes. Third, semantic overlaps across clusters and subclusters reveal that these research traditions are not conceptually isolated but are separated more by structure than by substance. Fourth, well-being remains marginal across the dominant thematic streams, even where sustainability, responsibility, and social value are discussed. These four findings provide the empirical foundation for the Discussion section, which interprets their implications and uses them to develop the Integrated Framework for Innovation and Well-being (IFIW).

## 5. Discussion

This section interprets the implications of these findings for innovation theory and develops the Integrated Framework for Innovation and Well-being (IFIW), with particular attention to framework fragmentation, the meso-level bridging role, semantic overlaps, and the limited integration of well-being.

### 5.1. Implications of the bibliometric analysis

A key finding confirmed by our analysis is the persistent fragmentation between the innovation and innovativeness research streams, often reflecting a divergence in the primary level of analysis. As detailed in the Result section (Focus level patterns and thematic trends in innovation and innovativeness frameworks), the innovation framework literature, represented by clusters such as IN 2 (Innovation Systems Analysis), IN 3 (Sustainable Innovation Management), and IN 11 (Social Innovation Theory and Framework Development), frequently adopts a macro-level, top-down perspective, focusing on systemic structures, policy interventions, and ecosystem-wide dynamics. Conversely, the innovativeness framework literature, exemplified by clusters like IS 1 (Organizational Innovativeness and Performance) and IS 5 (Technology Adoption Behavior), predominantly concentrates on the micro-level. These studies emphasize individual traits, cognitive factors, user behaviors, and firm-level capabilities, adopting a bottom-up view centered on the drivers and adoption of innovation. This bifurcation, while yielding deep insights within each domain, limits the development of holistic models that capture the interplay between individual actions and broader systemic outcomes.

Perhaps the most significant implication arising from our analysis is the confirmation of the underdeveloped yet crucial role of the meso-level in bridging this micro–macro divide. While meso-level perspectives were found to be generally underrepresented compared to micro and macro across the entire dataset, our analysis identified specific innovation subclusters where meso-level dynamics are central, including IN 4–3 (Innovation Ecosystem Dynamics, 74.5% meso focus), IN 5–5 (Network-Based Innovation and Knowledge Transfer, 73.8% meso focus), IN 8–2 (National Innovation Ecosystem Design, 69% meso focus), and IN 4–6 (Network Innovation Management, 68.1% meso focus). The core themes within these subclusters (inter-firm collaboration, value co-creation, alliance portfolio management, and network orchestration) show that the meso level is not simply an intermediate analytical layer but an active mechanism of translation and scaling. This interpretation is consistent with transition research showing that local innovation processes rarely scale in a linear way; rather, they evolve through interactions between niche-level experimentation and wider socio-technical environments, as Geels and Schot explain in their typology of transition pathways [76]. Smith and Raven [77] further clarify that such scaling depends on meso-level protective spaces that can shield, nurture, and eventually empower emerging innovations to influence broader regimes. In a similar vein, Klerkx and Aarts [78] show that innovation networks often require active orchestration by multiple champions to align actors and sustain innovation trajectories, while Klerkx and Leeuwis [79] demonstrate that innovation brokers play a strategic role in connecting fragmented actors and embedding innovations across system levels. Taken together, these studies reinforce our finding that the meso level is where user- and firm-level innovation signals are stabilized, coordinated, and amplified before influencing wider institutional and system-level change.

Furthermore, the semantic similarity analysis presented in the Result section (Semantic intersections between innovation and innovativeness) and detailed in our supplementary materials (S2 Table in S1 File) reveals substantial conceptual overlaps between the innovation and innovativeness domains, despite their structural separation. High similarity scores were observed between innovativeness clusters focused on organizational performance (IS 1) and innovation subclusters examining the innovation-performance nexus (IN 1–1) and strategic management (IN 1–5). Similarly, strong links exist between clusters centered on consumer/user innovativeness and technology adoption (IS 3, IS 5, IS 6) and innovation subclusters dealing with technology adoption models and consumer behavior (IN 6–6, IN 6–8). Connections were also found between Innovation Management and Market Adoption Strategies (IS 7) and Innovation Capabilities and Organizational Performance (IN 1–5), and between Innovation Management and Performance Outcomes (IS 2) and Innovation Implementation and Adoption Dynamics (IN 1–10). These semantic intersections strongly suggest that the division between the frameworks is more structural than conceptual. Core concepts like performance, strategy, adoption, user behavior, and organizational learning are inherently multi-level and relevant to both domains. This finding underscores the need for integrated frameworks that transcend the traditional boundaries and capture the continuous flow of influence from individual traits and behaviors through organizational processes to systemic outcomes.

The thematic streams identified in the Results also suggest a broader intellectual trajectory in the field. The earlier managerial stream is centered on internal capability building, where innovation is closely associated with organizational learning, culture, and performance. This interpretation is reinforced by Cohen and Levinthal’s concept of absorptive capacity [80], which highlights firms’ ability to recognize, assimilate, and apply external knowledge as a foundation for innovation, and by Crossan and Apaydin’s [81] synthesis of organizational innovation as a multidimensional construct linking leadership, process, and outcome. A later systemic turn broadens the unit of analysis from the single firm to ecosystems, networks, and institutional coordination, while a more recent user-centric stream shifts the focus toward individual adoption, digital interaction, and personal innovativeness. In this stream, Agarwal and Prasad’s conceptualization of personal innovativeness in information technology provides an important foundation for understanding why some individuals are more willing than others to engage with new digital tools [82]. These studies strengthen our interpretation that the field has evolved from internal capability development to inter-organizational coordination, to user-centered digital adoption, and finally toward more purpose-driven and socially oriented concerns. Importantly, this interpretation was not derived from isolated readings alone, but from triangulation across keyword patterns, highly cited publications, and the structural relations among clusters and subclusters.

Finally, our analysis confirms the significant gap concerning the integration of well-being within both innovation and innovativeness frameworks. While several clusters implicitly relate to broader innovation challenges—such as ethical design, sustainability transitions, and digital transformation, the explicit integration of well-being remains limited. Our analysis of the top 20 most cited articles (2,760 articles) across 138 subclusters within the innovation framework dataset revealed that only six articles explicitly mention well-being in their titles. Expanding the scope to include abstracts and author keywords increased this count to 366 out of 30,258 articles (only about 1.2%). At cluster level, Sustainable Innovation Management (IN 3), Social Innovation Theory and Framework Development (IN 11), Organizational Innovation Drivers and Performance Outcomes (IN 1) emerge as the most well-being-inclusive clusters. This aligns with previous findings in the literature that highlight the marginal treatment of well-being within innovation and innovativeness frameworks [9,10,35]. While concepts related to societal benefit or user experience appear implicitly in some clusters, such as those focused on social innovation (e.g., IN 11) or inclusive innovation (e.g., IN 11−2), well-being is rarely treated as an explicit, central objective or outcome measure within the core literature identified by our clustering. This omission persists despite growing recognition in policy and related academic fields of the importance of holistic progress metrics that extend beyond purely economic or technological indicators frameworks [9–11]. The marginalization of well-being within dominant innovation paradigms represents a major limitation, potentially leading to innovations that exacerbate inequalities or neglect crucial social and environmental dimensions. Addressing this gap is essential for aligning innovation efforts with broader societal goals and achieving truly sustainable development.

Taken together, these implications—the micro-macro fragmentation, the critical bridging role of the understudied meso-level, the underlying conceptual connections obscured by structural divides, and the neglect of well-being—highlight the need for a more unified, multi-level, and purpose-driven approach to understanding and fostering innovation. Building upon these insights, the following section proposes the Integrated Framework for Innovation and Well-being (IFIW) as a conceptual tool designed to address these challenges.

### 5.2. The integrated framework for innovation and well-being (IFIW)

To address the structural and thematic gaps identified in the preceding analysis, we propose the Integrated Framework for Innovation and Well-being (IFIW). The IFIW is designed as a conceptual tool for linking micro-, meso-, and macro-level innovation processes within a coherent structure while explicitly positioning well-being as a central priority. Rather than treating innovation as a linear sequence, the framework conceptualizes it as a multi-level and interactive process shaped by reciprocal influence across levels. To make the derivation of the framework transparent, we first translate the main empirical findings into eight guiding principles. As summarized in Table 4, each principle is linked to specific bibliometric results and qualitative findings, including focus-level patterns, meso-oriented subclusters, semantic intersections between innovation and innovativeness, thematic-stream interpretation, and the limited explicit integration of well-being. The subsequent subsections then elaborate these principles and discuss their theoretical and practical implications.

**Table 4:** Evidence-to-principle derivation of the Integrated Framework for Innovation and Well-being (IFIW).

Guiding principle	Main empirical level emphasized	Specific bibliometric and/or qualitative basis	Evidence-to-principle logic
**1. Interconnected levels and feedback loops**	Cross-level	Innovation clusters show strong macro orientation, while innovativeness clusters are strongly micro-oriented. Examples include macro-oriented innovation clusters such as **IN 2** (Innovation Systems Analysis), **IN 3** (Sustainable Innovation Management), and **IN 10** (Innovation Implementation Frameworks), and micro-oriented innovativeness clusters such as **IS 3** (User Adoption Factors of Personal Digital Augmentation Technologies), **IS 5** (Personal Innovativeness in Technology Adoption Behavior), and **IS 6** (Technology Acceptance Models in Human-Enhancement Systems and Healthcare Technologies). Semantic overlaps also connect technology adoption, organizational capability, and innovation-performance themes.	The observed separation between macro-oriented innovation research and micro-oriented innovativeness research motivates treating innovation as a reciprocal cross-level process rather than a one-directional sequence. Explicit feedback loops are therefore needed to connect bottom-up emergence from individuals and firms, meso-level coordination through networks and ecosystems, and top-down enablement through institutions and policy.
**2. Contextual adaptability and cultural sensitivity**	Cross-level	Context-sensitive themes appear in subclusters such as **IN 2−4** (Geographic Innovation Systems and Regional Development), **IN 8−6** (Helix Innovation System Models), **IN 8−10** (Cultural and Institutional Determinants of Cross-National Entrepreneurship), **IN 10−6** (Place-Based Innovation District Development), **IN 6−6** (Consumer Resistance and Adoption Patterns), and **IS 5** (Personal Innovativeness in Technology Adoption Behavior). Social innovation themes, especially **IN 11** and **IN 11−1**, also highlight community needs and social context.	Variation across regional, institutional, cultural, sectoral, and user contexts indicates that adaptability is a condition for effective cross-level integration. This principle ensures that innovation frameworks do not assume universal pathways of adoption, diffusion, or scaling, but remain responsive to local needs, cultural expectations, and institutional settings.
**3. Scalability through meso-level mechanisms**	Primarily meso; cross-level effect	Meso-oriented subclusters such as **IN 4–3** (Innovation Ecosystem Dynamics), **IN 5–5** (Network-Based Innovation and Knowledge Transfer), **IN 8–2** (National Innovation Ecosystem Design), and **IN 4–6** (Network Innovation Management) emphasize ecosystems, network-based knowledge transfer, ecosystem design, network management, orchestration, and inter-organizational coordination.	Meso-oriented subclusters indicate that ecosystems, networks, collaboration, brokerage, and orchestration mediate the movement from localized innovation to broader impact. The IFIW therefore positions the meso level as the main mechanism of scalability, where micro-level experimentation is translated into durable organizational, sectoral, and systemic change.
**4. Well-being as a cross-level priority**	Cross-level	Well-being is marginal across the dataset: only six of the top-cited articles explicitly mention well-being in titles, and only about 1.2% of innovation articles mention well-being in titles, abstracts, or keywords. Potential integration points appear in **IN 3** (Sustainable Innovation Management), **IN 11** (Social Innovation Theory and Framework Development), **IN 1** (Organizational Innovation Drivers and Performance Outcomes), **IN 1−1** (Innovation-Performance Nexus), and **IS 1** (Organizational Innovation Capability).	The marginal presence of well-being in the core innovation and innovativeness literature motivates its treatment as an explicit design objective and evaluation criterion across levels. This principle shifts the framework from innovation focused mainly on performance, adoption, or efficiency toward innovation oriented to holistic and enduring improvements for people and the planet.
**5. Ethical integrity and social equity**	Cross-level	Purpose-driven innovation, responsible innovation governance, emerging technology governance, digital transformation, user resistance, and social innovation themes highlight risks related to exclusion, bias, privacy, unequal access, and uneven distribution of innovation benefits. Relevant areas include **IN 12−1** (Responsible Innovation Governance), **IN 12−18** (Regulatory Frameworks for Emerging Technology Innovation), **IN 4−5** (Digital Business Transformation through Servitization), **IN 6−6** (Consumer Resistance and Adoption Patterns), and **IN 11−1** (Social Innovation).	Evidence from purpose-driven innovation, digital transformation, responsible innovation governance, and social innovation highlights risks of exclusion, bias, privacy loss, unequal access, and uneven benefit distribution. The IFIW therefore embeds ethics and equity as safeguards to ensure that innovation is judged not only by whether it creates value, but also by how responsibly that value is created, governed, and distributed.
**6. Environmental sustainability**	Primarily macro–meso	Sustainability-oriented clusters and subclusters, including **IN 3** (Sustainable Innovation Management), **IN 7** (Sustainable Business Model Innovation), **IN 3−4** (Sustainability-Oriented Innovation), **IN 3−6** (Innovation-Driven Environmental Sustainability in Energy Systems), and **IN 10−7** (Transdisciplinary Nature-Based Innovation for Sustainable Development), show growing attention to environmental transitions, circularity, and ecological resilience.	Sustainability-oriented clusters show that innovation can either support or undermine ecological resilience. The IFIW therefore treats environmental sustainability as a foundational criterion of innovation design, scaling, and evaluation, linking innovation to environmental stewardship and to the ecological conditions required for long-term societal well-being.
**7. Technology and digital transformation**	Cross-level	User-centric innovativeness clusters such as **IS 3**, **IS 5**, and **IS 6** overlap with innovation subclusters on digital adoption, digital transformation, and technology adoption models. Relevant innovation subclusters include **IN 4–5** (Digital Business Transformation through Servitization), **IN 6–6** (Consumer Resistance and Adoption Patterns), **IN 6–7** (Digital Technology Adoption), **IN 6–8** (Technology Adoption Models), and **IN 6–12** (Digital Technology Adoption and Implementation).	Digital transformation connects user adoption at the micro level, organizational readiness at the meso level, and infrastructure and governance at the macro level. This principle treats technology as a cross-level enabler that requires responsible coordination so that digital innovation remains human-centered, context-sensitive, ethically governed, and aligned with broader well-being and sustainability goals.
**8. Holistic evaluation and cross-level metrics**	Cross-level	Performance-oriented clusters such as **IN 1−1** (Innovation-Performance Nexus) and **IS 1** (Organizational Innovation Capability), evaluation-related subclusters such as **IN 9−3** (Surgical Innovation Evaluation) and **IN 9−6** (Performance-Based Innovation Assessment), and the well-being gap collectively show the limits of narrow innovation metrics.	Performance-oriented and evaluation-related clusters reveal the limits of narrow innovation metrics. The IFIW therefore requires assessment approaches that capture interconnected impacts across micro, meso, and macro levels by combining economic and technological indicators with measures of well-being, equity, environmental sustainability, collaboration quality, and long-term societal contribution.

#### 5.2.1. Addressing fragmentation and integrating levels.

To counter the fragmentation between micro-focused innovativeness research and macro-focused innovation research, the IFIW begins with principles that explicitly recognize the multi-level nature of innovation.

**1. Interconnected levels and feedback loops:** Innovation unfolds across micro (individual and firm), meso (organizational, network, and industry), and macro (systemic and societal) levels. Effective innovation therefore requires both coordination and feedback across these levels. Our analysis highlights the distinct emphasis of micro-level innovativeness clusters, such as IS 5 on technology adoption behavior, and macro-level innovation subclusters, such as IN 8−6 on regional policy. The IFIW interprets these levels as linked through bidirectional causal flows. **In bottom-up processes**, individual adoption choices, experimentation, and user insights are aggregated and validated through meso-level networks and platforms, which can then shape industry norms and broader societal change. **In top-down processes**, policies, regulations, and institutional priorities create the stability, legitimacy, and resources that enable meso-level ecosystems to support and incentivize innovation at the micro level. In this sense, the meso level is essential, as evidenced by clusters like IN 4−3 (Innovation Ecosystem Dynamics) and IN 5−5 (Network-Based Innovation and Knowledge Transfer), because it translates local innovation signals into organizational strategies and wider collective trajectories [65,83]. This role is often performed through collaborative platforms, intermediaries, innovation brokers, and network orchestrators that connect otherwise fragmented actors and strengthen cross-level alignment [78,79]. Multinational enterprises can also perform this bridging role by linking local innovation activity to wider global systems [84].
**2. Contextual adaptability and cultural sensitivity:** Innovation does not occur in a vacuum. Its success and impact depend on the social, economic, institutional, and cultural settings in which it is developed and implemented. The IFIW therefore emphasizes adaptability to local constraints, values, and available resources. This principle is reflected in clusters dealing with regional innovation systems, such as IN 8−6, and user adoption patterns, such as IN 6−6 and IS 5, which together suggest that innovation strategies must align technological solutions with community needs and cultural expectations [85,86]. Participatory approaches, including those found in urban resilience planning and social innovation initiatives related to IN 11, are especially important because they help connect top-down priorities with bottom-up realities [87].

Building on the central role of the meso level identified in our analysis, the next principle explains how these intermediate structures enable innovations to scale beyond local experimentation.

#### 5.2.2. Leveraging the meso-level for scalability.

The IFIW highlights the meso level not only as a bridging layer, but also as a mechanism through which innovation can achieve broader and more durable impact.

**3. Scalability through meso-level mechanisms:** For innovations to generate meaningful and widespread impact, they must move beyond isolated micro-level initiatives, such as individual creativity or pilot projects, and connect to broader system-level change. Our analysis strongly supports the view that this scaling process is mediated by the meso level. Subclusters focused on innovation ecosystems (IN 4−3), network-based knowledge transfer (IN 5−5), and national innovation ecosystem design (IN 8−2) show how diffusion through inter-organizational networks, platform-based coordination, and ecosystem orchestration can help innovations grow beyond their initial context. Structured environments such as living labs and testbeds further support this process by linking experimentation to wider implementation [88,89]. Scalability within the IFIW therefore requires leveraging meso-level structures while balancing standardization for efficiency with contextual adaptability for relevance [90]. From this perspective, scalability is not simply a matter of diffusion volume, but of how localized experimentation is protected, nurtured, and eventually strengthened enough to influence wider socio-technical arrangements [76,77].

Beyond structural coherence and scalability, innovation must also be guided by broader societal goals. The next principles therefore address well-being, ethics, and sustainability as core design priorities.

#### 5.2.3. Integrating purpose and responsibility.

Addressing the well-being gap identified in our analysis requires embedding social purpose and responsibility into the innovation process

**4. Well-being as a cross-level priority:** A central tenet of the IFIW is the explicit integration of well-being as both an innovation objective and an evaluation criterion across all levels. As confirmed by our analysis, well-being remains significantly underrepresented in the dominant themes of innovation and innovativeness literature, despite its recognized importance for sustainable and inclusive progress [9,10]. Although our analysis identified some references to well-being, particularly within Sustainable Innovation Management (IN 3), Social Innovation (IN 11), and Organizational Performance (IN 1−1, IS 1), it is rarely treated as a core organizing principle. The IFIW therefore advocates integrating well-being-related measures—such as subjective well-being, social cohesion, job satisfaction, and quality of life—alongside traditional economic and technological indicators [11]. This is consistent with broader international agendas such as the UN Sustainable Development Goals [12,13]. To support future empirical work, well-being should be understood as multidimensional, including physical, mental, financial, social, and capability-based aspects of human flourishing [35]. In practical terms, future studies could assess indicators such as life satisfaction, burnout and mental health, perceived financial security, inclusion and accessibility, trust and social cohesion, and opportunities for participation, learning, and meaningful contribution. Innovations such as remote working arrangements that seek to balance efficiency with employee mental health illustrate how well-being can guide both design and evaluation [91,92].
**5. Ethical integrity and social equity:** Innovation processes and outcomes should be guided by ethical reflection and a commitment to equity. The IFIW therefore calls for proactive attention to potential harms, unequal power relations, and barriers to access. These issues are especially important in fast-changing technological domains, as reflected in clusters dealing with responsible innovation governance (IN 12–1), regulatory frameworks for emerging technology innovation (IN 12–18), and digital business transformation (IN 4–5). Ethical governance mechanisms are needed to address risks related to data privacy, algorithmic bias, job displacement, and the digital divide [93–95]. Innovation should not only create value, but also distribute its benefits fairly so that marginalized groups are not excluded [95,96].
**6. Environmental sustainability:** Environmental sustainability is a foundational principle of the IFIW. This requires integrating ecological considerations throughout the innovation lifecycle, from design to implementation and disposal. Our analysis identified clusters specifically focused on Sustainability-Oriented Innovation (IN 3−4), Innovation-Driven Environmental Sustainability in Energy Systems (IN 3−6), and Transdisciplinary Nature-Based Innovation for Sustainable Development (IN 10−7). These studies highlight the importance of resource efficiency, pollution reduction, green technologies, and circular economy approaches [97,98]. A meaningful innovation framework must therefore ensure that innovation does not undermine the ecological systems on which long-term societal well-being depends.

The framework also requires principles that support implementation and assessment. The following section addresses digital transformation and holistic evaluation.

#### 5.2.4. Enabling and assessing integrated innovation.

The final two principles focus on the tools and evaluative approaches needed to implement innovation within an integrated, purpose-driven framework.

**7. Technology and digital transformation:** Digital technologies have become central to contemporary innovation and must therefore be integrated carefully within innovation frameworks. The IFIW acknowledges the transformative potential of digitalization but emphasizes that digital transformation should remain human-centered, context-sensitive, and system-aware. Clusters related to digital business transformation (IN 4–5), responsible innovation governance (IN 12–1), regulatory frameworks for emerging technology innovation (IN 12–18), and technology adoption (IS 3, IS 5, IN 6–6, IN 6–8) highlight the complexity of this challenge. Effective digital transformation requires alignment between technological capabilities, organizational readiness, user needs, digital literacy, and the broader institutional environment [99–103]. In this sense, digital transformation is both an outcome of innovativeness and a condition that can further stimulate innovativeness. Addressing adoption barriers and ethical concerns simultaneously is therefore essential if digital innovation is to remain inclusive and beneficial.
**8. Holistic evaluation and cross-level metrics:** The IFIW calls for evaluation approaches that move beyond narrow, single-level metrics such as firm profit or adoption rates. Innovation should instead be assessed in terms of its interconnected impacts across micro, meso, and macro levels. Evaluation should therefore combine conventional performance indicators with measures related to social equity, environmental sustainability, and well-being, drawing on clusters associated with performance (IN 1−1, IN 9−3, IN 9−6, IS 1) and value co-creation (IN 4−3) [2,11,104]. At the micro level, relevant indicators may include life satisfaction, job satisfaction, burnout, and digital stress; at the meso level, collaboration quality, employee retention, and organizational trust; and at the macro level, accessibility, inclusion, environmental quality, and community resilience. Multi-criteria assessment tools and long-term evaluation perspectives are particularly important because they allow innovation to be judged not only by short-term outputs, but by its broader societal contribution.

#### 5.2.5. Visualizing the framework: dynamics of cross-level influence.

**Fig 3:**
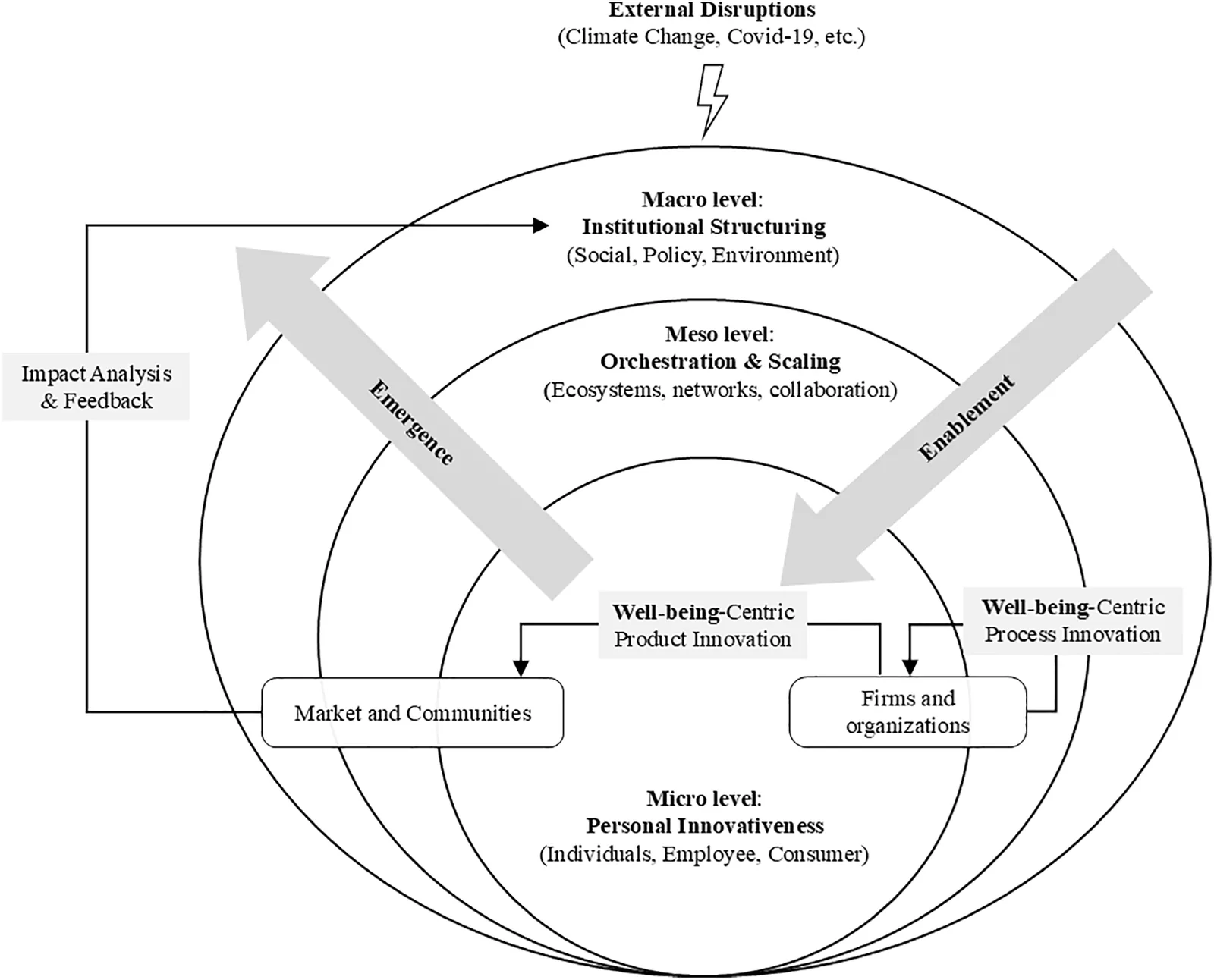
The Integrated Framework for Innovation and Well-being (IFIW). The model illustrates the dynamic interaction between analytical levels. The Micro level drives *Emergence* (bottom-up flow), where personal innovativeness creates new norms and value. The Meso level facilitates *Orchestration & Scaling*, acting as a bridge that translates individual insights into systemic outcomes. The Macro level provides *Enablement* (top-down flow) through institutional structuring, policy support, and resource allocation. A continuous feedback loop (left) ensures that market and community outcomes inform future policy and innovation cycles.

Fig 3 provides a visual synthesis of the IFIW by showing how innovation operates across interconnected levels under a shared well-being orientation. Rather than repeating the principles, the figure illustrates how they interact dynamically through directional influence and feedback.

At the center of the framework is the individual, reflecting the importance of human-centered design, personal innovativeness, and user-driven insight as foundations of innovation. Surrounding this core, the micro level represents individuals in their roles as consumers, citizens, and employees, whose needs, choices, and creative actions generate the initial signals that shape innovation processes. The meso level functions as the principal site of coordination and scaling. It captures organizational networks, ecosystems, partnerships, and intermediary structures through which micro-level signals are translated into broader organizational practices and collective trajectories. In this sense, the meso level acts as the bridge between local experimentation and broader system-level outcomes. The macro level represents the wider institutional and societal environment, including policies, regulatory structures, and sustainability-oriented priorities. This level provides the conditions that enable, constrain, and redirect innovation by shaping incentives, legitimacy, and strategic direction.

The arrows in the figure represent reciprocal flows across levels. Bottom-up dynamics show how individual behavior, user adoption, and local experimentation can influence organizational coordination and, over time, broader institutional change. Top-down dynamics show how macro-level priorities and regulatory frameworks shape the opportunities available to organizations and individuals. Continuous feedback loops ensure that innovation remains responsive to community needs, market outcomes, and changing societal conditions.

An important distinction highlighted by the framework is the shift from human-centered to well-being-centered innovation. Human-centered innovation focuses primarily on immediate user needs, usability, and experience [105,106]. By contrast, the IFIW broadens the evaluative scope of innovation to include longer-term and wider outcomes related to social cohesion, inclusion, sustainability, and environmental stewardship. In this view, well-being is treated not only as an outcome of innovation but also as a guiding condition for how innovation should be designed, scaled, and assessed. More fundamentally, well-being-centric innovation seeks holistic and enduring improvements for people and the planet, extending the focus of innovation beyond short-term individual benefits toward more sustainable and socially meaningful forms of progress.

By embedding well-being across micro, meso, and macro levels, the IFIW links individual creativity, organizational coordination, and systemic priorities within a single framework. This cross-level alignment encourages innovation strategies that move beyond narrow economic goals and toward more inclusive, sustainable, and socially meaningful forms of progress grounded in both human flourishing and environmental stewardship [107,108]. It also has practical organizational relevance. Research shows that practices supporting employee well-being and broader societal contribution can strengthen consumer trust and loyalty [109–112], while internally they can also support creativity, knowledge sharing, and innovative behavior [113–116]. The figure therefore serves not merely as an illustration, but as a visual summary of how the framework aligns innovation processes with holistic and enduring improvements for people and the planet.

### 5.3. Practical implications

The Integrated Framework for Innovation and Well-being (IFIW) offers a structured and actionable approach for different stakeholder groups to integrate innovation efforts with the goal of enhancing societal well-being. By explicitly connecting micro, meso, and macro levels, the framework moves beyond theoretical integration to provide concrete implementation guidance.

**Implications for Organizational and Innovation Managers** Organizational leaders can utilize the IFIW to move beyond siloed R&D and strategically manage innovation across internal teams (Micro) and external networks (Meso). First, managers should aim for balancing ambidexterity by using the IFIW’s multi-level perspective to bridge exploration and exploitation. This involves fostering exploration at the micro-level through individual creativity and experimentation, while simultaneously pursuing exploitation at the meso-level by leveraging established networks and supply chains. To achieve this, firms should focus on building meso-level capabilities—specifically absorptive capacity, organizational agility, and networked collaboration—to strategically integrate well-being-oriented innovations into their business models [19–21]. Second, leaders must focus on integrating well-being metrics directly into business models. Rather than viewing well-being merely as a peripheral Corporate Social Responsibility (CSR) add-on, firms should recognize that integrating sustainability and social responsibility directly into innovation practices strengthens consumer trust and enhances brand loyalty [107–110]. Finally, a strategy for meso-level collaboration is essential; leaders should prioritize “bridging” connections with non-traditional partners to increase absorptive capacity and ensure innovations meet real societal needs [14,29].

**Implications for Policymakers and Public Institutions** The IFIW provides a lens for designing effective, multi-level policies that address the structural gaps identified in the innovation landscape. A primary shift involves targeted meso-level funding. Policymakers should move away from solely funding individual firms (Micro) or broad national projects (Macro) and instead create mandated funding for Meso-level Consortia to collaboratively tackle well-being challenges. Additionally, policy must ensure systemic regulatory alignment. Macro-level regulations must be adaptable enough to foster rather than stifle micro-level innovations; policymakers can leverage mechanisms such as living labs and testbeds, which provide structured environments to manage the scaling of innovations from local experimentation to wider systemic adoption [86,87]. By adopting frameworks that encourage cross-sector collaboration and participatory governance, policymakers can align innovation with societal priorities, such as sustainability and social inclusion [22,31]. Crucially, the IFIW advocates for a policy evaluation focus that utilizes specific instruments like innovation incentives and open-government initiatives to foster transparency and collaboration [7,16].

**Implications for Academic Institutions and Researchers** The IFIW serves as a structured theoretical foundation, guiding future research to address the observed disciplinary and methodological fragmentation. Academic institutions should prioritize curriculum development that establishes interdisciplinary programs teaching systems thinking alongside traditional innovation concepts. This prepares future professionals to manage the multi-level dynamics inherent in the IFIW. Regarding the research agenda, there is a pressing need for advancing measurement methodologies; the framework encourages scholars to investigate the multi-level dynamics of innovation systematically. Researchers are encouraged to develop robust metrics capable of capturing the complex interactions among individual behaviors, organizational strategies, and systemic innovation outcomes [33–35]. Finally, the field requires empirical validation; specifically, there is a critical need to operationalize subjective well-being (SWB) as a core indicator of innovation impact, analyzing it alongside traditional economic metrics to ensure a holistic assessment of progress [11].

### 5.4. Methodological Limitations

While this study utilizes the Web of Science (WoS) Core Collection to ensure high metadata consistency for bibliometric clustering, we acknowledge that relying on a single database constitutes a limitation regarding the comprehensiveness of the sample. As highlighted in comparative scientometric studies, databases such as Scopus and Dimensions offer wider coverage of source titles, particularly regarding conference proceedings, book chapters, and non-English regional literature [44,117]. Consequently, our analysis may underrepresent specific niche debates or “grey literature” (theses, reports) that are more visible in broader repositories like Google Scholar or Dimensions [118,119].

However, the choice of WoS was a deliberate methodological decision prioritizing selectivity and link quality over raw comprehensiveness. As Visser et al. (2021) argue, the value of a bibliographic source depends on the research purpose; while Dimensions offers vast coverage, it suffers from significant issues with missing or incomplete citation links, which can distort the clustering algorithms [44]. For constructing a robust theoretical framework (IFIW), we required a dataset of “certified knowledge” with stable citation networks. WoS provides this rigorous filtering mechanism, ensuring that the identified clusters represent established scholarly consensus rather than transient or peripheral publications.

Furthermore, while Scopus and Dimensions are larger, evidence suggests that the “core” structural knowledge of a field is highly correlated across databases. Martín-Martín et al. (2018) found that despite the unique coverage of Google Scholar, Spearman correlations between citation counts in WoS and broader databases remain high (0.78–0.99) [119]. This suggests that the influential “backbone” of the innovation and innovativeness literature identified in our study is likely stable, even if peripheral documents were excluded. Nevertheless, we acknowledge that both WoS and Scopus share inherent biases toward Natural Sciences and English-language outputs [117]. Future research could therefore extend the validity of the IFIW by applying our search string to Scopus or Dimensions to capture regional innovation practices and practitioner-oriented literature, thereby testing the framework’s applicability in more diverse and less formal contexts.

Finally, our assessment of well-being integration relies on keyword occurrences within titles, abstracts, and author keywords. We acknowledge that this metadata-based approach may yield a conservative estimate, as it excludes articles that might discuss well-being in the full text (e.g., in the discussion or implications sections) without highlighting it in the metadata. Future research employing full-text natural language processing could provide a more granular measurement of this gap.

Because the IFIW is proposed as a conceptual framework, its empirical value depends on future validation across different innovation contexts, stakeholder groups, and methodological designs. The following Future Research Agenda therefore outlines concrete pathways for testing and refining the framework.

## 6. Future research agenda

To further validate, refine, and extend the Integrated Framework for Innovation and Well-being (IFIW), future research should examine the framework through complementary empirical strategies. First, comparative case studies could apply the IFIW across different innovation contexts, such as digital transformation, sustainability transitions, healthcare innovation, mobility systems, or regional innovation policy, to assess whether the framework explains how micro-level innovativeness is translated through meso-level structures into macro-level outcomes. Second, expert evaluation, including Delphi panels or structured stakeholder workshops with researchers, policymakers, innovation managers, and civil-society actors, could assess the clarity, completeness, and practical relevance of the eight principles. Third, application-based validation could use the IFIW as an analytical template in real innovation projects and compare framework-based diagnoses with observed outcomes, such as adoption, collaboration quality, equity, sustainability performance, and well-being indicators. These strategies would help move the IFIW from a conceptual model toward an empirically refined and practically usable framework. Building on these validation pathways, we propose the following targeted research avenues:

**Empirical validation of cross-level dynamics**. Future research should examine the bidirectional causal flows proposed in the IFIW through comparative and longitudinal case studies. Specifically, studies should investigate how bottom-up personal innovativeness, user adoption, and local experimentation at the micro level are translated through meso-level structures, such as ecosystems, innovation hubs, industry consortia, and digital platforms, into macro-level institutional or societal change. Conversely, research should also examine how macro-level policies, regulations, and funding instruments enable or constrain meso-level coordination and micro-level innovation behavior.

**Deepening Meso-Level Understanding**: Given our finding that the meso-level is the critical “missing link,” researchers should investigate specific bridging mechanisms—such as digital platforms, innovation hubs, and industry consortia. Comparative analysis of how these structures facilitate (or block) the translation of individual creativity into systemic impact is urgently needed.

**Operationalizing Well-being Metrics.** Addressing the marginalization of well-being requires more than theoretical advocacy. We call for the development and standardization of well-being-centric KPIs that can be used alongside traditional economic metrics. This includes piloting frameworks that explicitly measure subjective well-being (SWB) and social equity as primary innovation outcomes. At the micro level, such indicators may include life satisfaction, job satisfaction, burnout, and digital stress; at the meso level, collaboration quality, employee retention, and organizational trust; and at the macro level, accessibility, inclusion, environmental quality, and community resilience.

**Longitudinal Analysis of Thematic Evolution.** Building on our qualitative analysis of evolving research streams, longitudinal studies should track if and how the “Managerial Core” of innovation is integrating with “Purpose-Driven” discourses over time. Are firms truly adopting societal purpose, or does it remain a peripheral CSR activity?

**Testing the Framework in Diverse Contexts.** Recognizing the potential bias of our Web of Science dataset, future research should apply the IFIW principles in different innovation contexts (e.g., Western and non-Western). Investigating how these principles manifest in regions with different institutional structures will be crucial for establishing the framework’s global validity.

**Governing Digital Transformation via IFIW.** Finally, researchers should apply the IFIW to emerging technologies (AI, IoT). Specifically, how can the framework’s emphasis on “ethical integrity” and “user-centricity” guide the governance of algorithmic systems to prevent bias and ensure digital equity?

Addressing these questions will transition the IFIW from a conceptual model to an actionable toolkit for designing resilient, equitable, and well-being-oriented innovation ecosystems.

## 7. Conclusion

This study addressed critical gaps within innovation and innovativeness research by mapping the fragmented landscape of existing frameworks through a large-scale bibliometric analysis complemented by a qualitative analysis of evolving thematic streams. Our findings highlighted the underutilization of meso-level mechanisms for bridging micro- and macro-level dynamics and confirmed the significant marginalization of well-being as an explicit goal. Our qualitative analysis highlighted a clear evolution in the field, moving from firm-centric capabilities to purpose-driven societal outcomes, yet these research streams remain largely disconnected.

In response, we proposed the Integrated Framework for Innovation and Well-being (IFIW). Defined by a dynamic Micro-Meso-Macro architecture and underpinned by eight guiding principles, the IFIW offers a unified conceptual tool. Crucially, it articulates the bidirectional dynamics of innovation: facilitating bottom-up emergence through user-driven creativity and ensuring top-down enablement via supportive institutional structures. While our reliance on Web of Science data prioritizes certified scholarly knowledge over broader grey literature, the resulting framework provides a robust, evidence-based foundation for future research. By systematically integrating individual innovativeness, network dynamics, and systemic goals—while positioning well-being as a foundational cross-level priority—the IFIW moves beyond fragmented perspectives. It offers holistic, practical guidance for designing innovation ecosystems that are inclusive, resilient, and sustainable.

## Supporting information

S1 File Supplementary materials (S1 Table and S2 Table). (DOCX)
