# Supplementary material for: Bridging the gaps in innovation research: A bibliometric analysis and multi-level framework integrating meso-perspectives and well-being
Source: PLoS One. 2026 Aug 24;21(8):e0356731. doi: 10.1371/journal.pone.0356731 (PMC13502657; doi:10.1371/journal.pone.0356731)
Supplement: Supplementary file 1 [file pone.0356731.s001.docx]

**Supplementary materials**

**S1 Table. Innovation framework research subclusters with their number of articles (N), average publication year (APY), and level percentage (micro, meso, macro).**

| **ID** | Subcluster Name | N | APY | Micro | Meso | Macro |
| --- | --- | --- | --- | --- | --- | --- |
| IN 1-1 | Innovation-Performance Nexus | 684 | 2015.5 | 37% | 19% | 29% |
| IN 1-2 | Service-Dominant Logic Innovation Framework | 592 | 2019.6 | 56% | 46% | 41% |
| IN 1-3 | Theoretical Frameworks for Organizational Creativity | 435 | 2017.3 | 42% | 15% | 34% |
| IN 1-4 | Innovation Performance Measurement | 383 | 2013.5 | 41% | 22% | 27% |
| IN 1-5 | Innovation Capabilities and Organizational Performance | 288 | 2016.3 | 23% | 27% | 36% |
| IN 1-6 | Collaborative Innovation Models | 257 | 2018.0 | 49% | 33% | 40% |
| IN 1-7 | Quality Management and Innovation Performance | 219 | 2017.2 | 33% | 21% | 30% |
| IN 1-8 | Service Innovation Management Frameworks | 215 | 2015.9 | 40% | 27% | 34% |
| IN 1-9 | Organizational Culture and Innovation | 198 | 2017.4 | 32% | 11% | 26% |
| IN 1-10 | Innovation Implementation and Adoption Dynamics | 163 | 2016.0 | 51% | 18% | 32% |
| IN 1-11 | Tourism Service Innovation Management | 156 | 2018.4 | 29% | 26% | 41% |
| IN 1-12 | Knowledge Management Systems' Impact on Organizational Performance and Innovation | 118 | 2015.9 | 25% | 28% | 35% |
| IN 1-13 | Performance Measurement Frameworks in Innovation Management | 536 | 2016.6 | 32% | 25% | 40% |
| IN 2-1 | Methodological Frameworks for Studying Innovation-Driven Transitions | 882 | 2019.7 | 17% | 26% | 90% |
| IN 2-2 | Innovation Systems Analysis Frameworks | 807 | 2018.1 | 14% | 32% | 79% |
| IN 2-3 | Agricultural Innovation Adoption | 601 | 2019.0 | 28% | 40% | 76% |
| IN 2-4 | Geographic Innovation Systems and Regional Development | 443 | 2017.8 | 11% | 33% | 84% |
| IN 2-5 | Innovation Systems and Capability Development | 417 | 2016.2 | 11% | 27% | 76% |
| IN 2-6 | Sustainability Experimentation | 237 | 2018.5 | 15% | 29% | 84% |
| IN 2-7 | Innovation Process Frameworks and Models | 549 | 2017.9 | 15% | 27% | 79% |
| IN 3-1 | Innovation in Sustainable Supply Chain Management | 490 | 2019.9 | 25% | 31% | 77% |
| IN 3-2 | Green Innovation Management and Strategy | 459 | 2021.4 | 21% | 18% | 72% |
| IN 3-3 | Environmental Innovation and Regulatory Impact | 373 | 2021.8 | 6% | 15% | 86% |
| IN 3-4 | Sustainability-Oriented Innovation | 366 | 2019.5 | 21% | 27% | 84% |
| IN 3-5 | Eco-Innovation Management | 358 | 2018.4 | 18% | 22% | 79% |
| IN 3-6 | Innovation-Driven Environmental Sustainability in Energy Systems | 311 | 2022.3 | 8% | 23% | 84% |
| IN 3-7 | Strategic CSR as an Innovation Driver | 230 | 2019.0 | 26% | 32% | 86% |
| IN 3-8 | Green Product Innovation Management | 179 | 2018.0 | 38% | 34% | 75% |
| IN 3-9 | Environmental Innovation Management | 369 | 2019.2 | 27% | 25% | 78% |
| IN 4-1 | Dynamic Capabilities | 664 | 2017.5 | 24% | 29% | 35% |
| IN 4-2 | Digital Architecture and Innovation Management | 569 | 2021.3 | 30% | 28% | 38% |
| IN 4-3 | Innovation Ecosystem Dynamics | 538 | 2020.1 | 26% | 75% | 45% |
| IN 4-4 | Organizational Ambidexterity | 292 | 2018.6 | 26% | 23% | 34% |
| IN 4-5 | Digital Business Transformation through Servitization | 189 | 2021.7 | 42% | 49% | 26% |
| IN 4-6 | Network Innovation Management | 141 | 2019.8 | 33% | 64% | 44% |
| IN 4-7 | Innovation-Driven International Business Strategy | 89 | 2019.3 | 17% | 42% | 74% |
| IN 4-8 | Innovation-Driven Organizational Resilience | 86 | 2020.0 | 35% | 38% | 35% |
| IN 4-9 | Innovation Framework Design and Implementation | 345 | 2017.3 | 28% | 33% | 41% |
| IN 5-1 | Open Innovation Management | 399 | 2019.4 | 23% | 40% | 33% |
| IN 5-2 | Organizational Knowledge Management Frameworks | 286 | 2016.1 | 24% | 31% | 37% |
| IN 5-3 | Knowledge Management Frameworks for Organizational Innovation | 226 | 2014.8 | 31% | 33% | 50% |
| IN 5-4 | Knowledge Transfer Mechanisms | 195 | 2016.5 | 18% | 36% | 61% |
| IN 5-5 | Network-Based Innovation and Knowledge Transfer | 149 | 2015.8 | 22% | 74% | 46% |
| IN 5-6 | Collaborative Innovation Management | 147 | 2016.8 | 52% | 48% | 30% |
| IN 5-7 | Strategic Management of Intellectual Property and Innovation Commercialization | 143 | 2016.2 | 13% | 21% | 31% |
| IN 5-8 | Boundary Objects in Knowledge Management for Innovation | 129 | 2015.7 | 29% | 46% | 37% |
| IN 5-9 | R&D Cooperation | 118 | 2016.8 | 23% | 38% | 48% |
| IN 5-10 | Strategic Innovation through Coopetition | 102 | 2019.0 | 19% | 45% | 34% |
| IN 5-11 | Organizational Knowledge Integration and Learning | 83 | 2015.3 | 13% | 43% | 31% |
| IN 5-12 | Open Source Software Innovation Management | 73 | 2016.2 | 33% | 36% | 47% |
| IN 5-13 | Knowledge-Based Innovation Management | 305 | 2016.8 | 25% | 32% | 44% |
| IN 6-1 | TOE Framework and Technology Adoption | 402 | 2018.3 | 41% | 15% | 42% |
| IN 6-2 | Industry 4.0 Implementation | 289 | 2021.2 | 32% | 37% | 61% |
| IN 6-3 | Blockchain Adoption and Implementation | 228 | 2021.6 | 36% | 35% | 57% |
| IN 6-4 | Innovation Adoption Theory | 210 | 2016.2 | 44% | 21% | 47% |
| IN 6-5 | Construction Industry Innovation Management | 193 | 2016.4 | 24% | 30% | 43% |
| IN 6-6 | Consumer Resistance and Adoption Patterns | 167 | 2020.1 | 80% | 19% | 56% |
| IN 6-7 | Digital Technology Adoption | 160 | 2021.9 | 44% | 23% | 58% |
| IN 6-8 | Technology Adoption Models | 106 | 2016.6 | 63% | 17% | 42% |
| IN 6-9 | Digital Technology Adoption | 97 | 2020.3 | 36% | 27% | 37% |
| IN 6-10 | Technology Adoption Dynamics in Advanced Manufacturing Innovation | 75 | 2019.4 | 52% | 27% | 43% |
| IN 6-11 | Technology Adoption and Organizational Change | 69 | 2013.3 | 39% | 28% | 43% |
| IN 6-12 | Digital Technology Adoption and Implementation | 64 | 2020.4 | 27% | 25% | 52% |
| IN 6-13 | Innovation Adoption Models | 259 | 2016.3 | 45% | 32% | 44% |
| IN 7-1 | Business Model Innovation and Evolution | 607 | 2019.8 | 30% | 27% | 35% |
| IN 7-2 | Business Model Innovation for Circular Economy | 554 | 2021.5 | 25% | 38% | 76% |
| IN 7-3 | Sustainable Business Model Innovation | 384 | 2020.7 | 25% | 33% | 88% |
| IN 7-4 | Product Service Systems (PSS) | 149 | 2018.2 | 50% | 27% | 55% |
| IN 7-5 | Platform-Based Business Model Innovation | 131 | 2020.7 | 46% | 26% | 58% |
| IN 7-6 | Disruptive Innovation | 216 | 2019.2 | 26% | 33% | 58% |
| IN 8-1 | University-Industry Innovation Partnerships | 307 | 2017.4 | 21% | 59% | 62% |
| IN 8-2 | National Innovation Ecosystem Design | 261 | 2020.8 | 22% | 69% | 78% |
| IN 8-3 | Academic Innovation Commercialization | 184 | 2018.2 | 21% | 38% | 68% |
| IN 8-4 | Business Incubator Performance Assessment | 133 | 2017.5 | 17% | 40% | 71% |
| IN 8-5 | International Corporate Entrepreneurship | 120 | 2017.1 | 32% | 12% | 48% |
| IN 8-6 | Helix Innovation System Models | 120 | 2019.9 | 9% | 58% | 83% |
| IN 8-7 | Entrepreneurship Research Methodologies | 106 | 2018.4 | 24% | 28% | 47% |
| IN 8-8 | Systematic Knowledge Integration in Innovation Research | 91 | 2021.3 | 34% | 19% | 64% |
| IN 8-9 | Theoretical Frameworks for Innovation | 74 | 2015.9 | 23% | 49% | 74% |
| IN 8-10 | Cultural and Institutional Determinants of Cross-National Entrepreneurship | 70 | 2017.1 | 36% | 19% | 66% |
| IN 8-11 | Entrepreneurial Innovation Dynamics | 60 | 2017.7 | 22% | 20% | 75% |
| IN 8-12 | Technology Transfer and Commercialization Frameworks | 58 | 2012.9 | 24% | 29% | 66% |
| IN 8-13 | Digital Innovation and Entrepreneurship Transformation | 55 | 2019.5 | 35% | 36% | 65% |
| IN 8-14 | Social and Knowledge Dynamics in Innovation Success | 225 | 2016.9 | 20% | 29% | 63% |
| IN 9-1 | Implementation Science Frameworks | 188 | 2020.9 | 47% | 36% | 45% |
| IN 9-2 | Innovation Implementation Frameworks | 179 | 2016.5 | 35% | 32% | 58% |
| IN 9-3 | Surgical Innovation Evaluation | 145 | 2018.9 | 10% | 17% | 37% |
| IN 9-4 | Healthcare Innovation Implementation Complexity | 144 | 2019.8 | 42% | 38% | 67% |
| IN 9-5 | Implementation Science Frameworks | 113 | 2018.8 | 40% | 39% | 57% |
| IN 9-6 | Performance-Based Innovation Assessment | 88 | 2019.6 | 24% | 19% | 56% |
| IN 9-7 | Healthcare Digital Transformation Management | 57 | 2018.9 | 51% | 30% | 60% |
| IN 9-8 | Innovation Diffusion | 55 | 2014.9 | 25% | 45% | 76% |
| IN 9-9 | Innovation Implementation Frameworks | 53 | 2017.2 | 30% | 26% | 55% |
| IN 9-10 | Healthcare Innovation Implementation | 53 | 2016.5 | 40% | 34% | 55% |
| IN 9-11 | Regulatory Innovation in Emerging Biomedical Technologies | 49 | 2018.1 | 18% | 31% | 76% |
| IN 9-12 | Healthcare Delivery Innovation Implementation | 47 | 2017.3 | 23% | 26% | 49% |
| IN 9-13 | Healthcare Innovation Implementation | 242 | 2017.8 | 29% | 31% | 58% |
| IN 10-1 | Smart City Implementation Frameworks | 266 | 2020.2 | 15% | 46% | 78% |
| IN 10-2 | Public Sector Innovation Management and Governance | 218 | 2019.3 | 24% | 37% | 68% |
| IN 10-3 | Digital Government Innovation | 122 | 2019.7 | 27% | 34% | 94% |
| IN 10-4 | Living Lab Methodologies | 112 | 2019.8 | 39% | 40% | 59% |
| IN 10-5 | Public Procurement Innovation | 96 | 2019.0 | 18% | 31% | 79% |
| IN 10-6 | Place-Based Innovation District Development | 81 | 2017.5 | 5% | 42% | 72% |
| IN 10-7 | Transdisciplinary Nature-Based Innovation for Sustainable Development | 54 | 2018.7 | 4% | 56% | 80% |
| IN 10-8 | Policy Innovation Diffusion | 50 | 2018.0 | 22% | 18% | 98% |
| IN 10-9 | Urban Resilience Innovation Systems | 50 | 2019.0 | 16% | 34% | 98% |
| IN 10-10 | Institutional Innovation Systems | 250 | 2017.9 | 20% | 29% | 75% |
| IN 11-1 | Social Innovation | 171 | 2020.1 | 17% | 44% | 96% |
| IN 11-2 | Inclusive Innovation for Bottom of the Pyramid Markets | 144 | 2019.6 | 27% | 28% | 72% |
| IN 11-3 | Social Innovation Implementation | 140 | 2019.9 | 22% | 32% | 93% |
| IN 11-4 | Social Enterprise Innovation Models and Strategies | 110 | 2020.0 | 25% | 32% | 95% |
| IN 11-5 | Frugal Innovation | 109 | 2019.6 | 42% | 20% | 69% |
| IN 11-6 | Network-Enabled Social Innovation Systems | 50 | 2019.2 | 18% | 54% | 96% |
| IN 11-7 | Institutional Innovation Frameworks | 48 | 2019.5 | 6% | 29% | 96% |
| IN 11-8 | Theoretical Frameworks for Innovation Management | 239 | 2018.3 | 26% | 44% | 71% |
| IN 12-1 | Responsible Innovation Governance | 559 | 2019.7 | 19% | 24% | 75% |
| IN 12-2 | Methodological Frameworks for Emerging Technology | 342 | 2017.8 | 19% | 35% | 48% |
| IN 12-3 | Geographic and Network Dynamics in Innovation Diffusion | 289 | 2016.9 | 11% | 58% | 76% |
| IN 12-4 | Innovation Economics and Market Dynamics | 287 | 2017.2 | 16% | 11% | 54% |
| IN 12-5 | Design Thinking | 238 | 2020.0 | 43% | 26% | 30% |
| IN 12-6 | National Innovation Systems Performance | 189 | 2017.8 | 11% | 32% | 76% |
| IN 12-7 | Digital-Enabled Product-Service Innovation | 137 | 2020.6 | 63% | 34% | 22% |
| IN 12-8 | Innovation Management in Family Businesses | 91 | 2019.6 | 15% | 19% | 32% |
| IN 12-9 | Agent-Based Modeling for Environmental Innovation Diffusion | 86 | 2016.8 | 47% | 30% | 62% |
| IN 12-10 | Effectiveness of Public Innovation Support Mechanisms | 84 | 2016.4 | 8% | 13% | 85% |
| IN 12-11 | Educational Technology Innovation Implementation | 77 | 2018.0 | 27% | 27% | 53% |
| IN 12-12 | Innovation Diffusion Models and Patterns | 72 | 2016.8 | 58% | 26% | 42% |
| IN 12-13 | Innovation Governance and Knowledge Protection Systems | 69 | 2017.2 | 19% | 13% | 70% |
| IN 12-14 | Innovation Management Frameworks | 60 | 2017.0 | 43% | 25% | 30% |
| IN 12-15 | Manufacturing Location Strategy and Innovation Capabilities | 59 | 2016.5 | 25% | 25% | 63% |
| IN 12-16 | Educational Innovation Frameworks | 58 | 2021.3 | 28% | 19% | 52% |
| IN 12-17 | Sustainable Process Innovation Methodologies | 54 | 2018.2 | 19% | 26% | 63% |
| IN 12-18 | Regulatory Frameworks for Emerging Technology Innovation | 52 | 2020.4 | 27% | 27% | 77% |
| IN 12-19 | Systemic Innovation Frameworks for Sustainable Development | 52 | 2017.7 | 21% | 23% | 87% |
| IN 12-20 | Integrated Design Frameworks for Systematic Innovation | 49 | 2017.6 | 51% | 35% | 14% |
| IN 12-21 | Cross-Border Innovation Systems | 47 | 2014.6 | 17% | 62% | 81% |
| IN 12-22 | Innovation Risk and Adoption Dynamics | 46 | 2015.9 | 24% | 17% | 59% |
| IN 12-23 | Institutional Transformation and Innovation Assessment | 731 | 2017.9 | 23% | 35% | 57% |

**S2 Table. High-similarity pairs between innovation subclusters and innovativeness clusters based on cosine similarity values exceeding the third quartile threshold (0.75), sorted in descending order.**

| Innovation subclusters | Innovativeness clusters | Cosine Similarity value |
| --- | --- | --- |
| IN 1-1 | IS 1 | 0.97252285 |
| IN 1-1 | IS 7 | 0.95182025 |
| IN 6-8 | IS 6 | 0.9394939 |
| IN 6-6 | IS 5 | 0.93551755 |
| IN 1-5 | IS 1 | 0.933711 |
| IN 6-1 | IS 4 | 0.9319029 |
| IN 1-10 | IS 2 | 0.93068403 |
| IN 6-6 | IS 3 | 0.9274983 |
| IN 6-6 | IS 6 | 0.9222425 |
| IN 1-1 | IS 2 | 0.9210001 |
| IN 1-10 | IS 8 | 0.91998595 |
| IN 6-8 | IS 5 | 0.9194288 |
| IN 1-5 | IS 7 | 0.9167501 |
| IN 6-6 | IS 8 | 0.9116886 |
| IN 6-8 | IS 8 | 0.91017914 |
| IN 1-10 | IS 7 | 0.90837 |
| IN 1-10 | IS 1 | 0.9027077 |
| IN 1-9 | IS 1 | 0.901368 |
| IN 1-99 | IS 1 | 0.9009421 |
| IN 4-1 | IS 1 | 0.8915062 |
| IN 6-7 | IS 4 | 0.8907347 |
| IN 6-8 | IS 3 | 0.8861038 |
| IN 4-4 | IS 1 | 0.8850261 |
| IN 1-4 | IS 1 | 0.8847246 |
| IN 1-4 | IS 7 | 0.8846795 |
| IN 1-1 | IS 8 | 0.880587 |
| IN 1-99 | IS 7 | 0.88011813 |
| IN 6-8 | IS 4 | 0.87759334 |
| IN 6-4 | IS 4 | 0.8767769 |
| IN 6-99 | IS 4 | 0.87410057 |
| IN 1-99 | IS 2 | 0.8733852 |
| IN 4-1 | IS 7 | 0.87336904 |
| IN 1-5 | IS 2 | 0.8727829 |
| IN 6-4 | IS 8 | 0.86950374 |
| IN 1-7 | IS 1 | 0.8633796 |
| IN 1-4 | IS 2 | 0.86189604 |
| IN 5-9 | IS 7 | 0.86080766 |
| IN 5-2 | IS 1 | 0.8602501 |
| IN 4-4 | IS 7 | 0.85927916 |
| IN 1-5 | IS 4 | 0.8572968 |
| IN 6-11 | IS 4 | 0.85666037 |
| IN 5-9 | IS 1 | 0.8547012 |
| IN 1-3 | IS 1 | 0.8525736 |
| IN 1-5 | IS 8 | 0.8518406 |
| IN 1-9 | IS 7 | 0.85127795 |
| IN 5-6 | IS 1 | 0.85067856 |
| IN 5-4 | IS 1 | 0.84976506 |
| IN 1-10 | IS 4 | 0.8489462 |
| IN 8-5 | IS 1 | 0.8483438 |
| IN 6-99 | IS 8 | 0.8482993 |
| IN 1-1 | IS 4 | 0.84660244 |
| IN 1-9 | IS 2 | 0.8441794 |
| IN 8-5 | IS 7 | 0.8433647 |
| IN 6-4 | IS 6 | 0.8423012 |
| IN 8-99 | IS 7 | 0.84136 |
| IN 4-99 | IS 7 | 0.8373622 |
| IN 1-6 | IS 2 | 0.83724403 |
| IN 5-11 | IS 7 | 0.8368988 |
| IN 1-8 | IS 2 | 0.83609194 |
| IN 5-6 | IS 7 | 0.83600944 |
| IN 6-1 | IS 8 | 0.83511233 |
| IN 1-8 | IS 1 | 0.8314333 |
| IN 5-11 | IS 1 | 0.83061016 |
| IN 5-4 | IS 7 | 0.83052427 |
| IN 5-6 | IS 2 | 0.8304677 |
| IN 8-99 | IS 1 | 0.82996404 |
| IN 6-6 | IS 4 | 0.82917434 |
| IN 4-99 | IS 1 | 0.82769156 |
| IN 1-3 | IS 2 | 0.82644916 |
| IN 4-4 | IS 2 | 0.82581985 |
| IN 1-99 | IS 8 | 0.8254727 |
| IN 4-1 | IS 2 | 0.82525384 |
| IN 5-99 | IS 1 | 0.82361794 |
| IN 1-7 | IS 2 | 0.82285786 |
| IN 5-2 | IS 7 | 0.8218876 |
| IN 8-8 | IS 8 | 0.8214551 |
| IN 6-99 | IS 2 | 0.8211304 |
| IN 6-99 | IS 7 | 0.82109976 |
| IN 8-8 | IS 7 | 0.82089657 |
| IN 6-11 | IS 8 | 0.8207944 |
| IN 5-1 | IS 7 | 0.8204579 |
| IN 8-8 | IS 1 | 0.819626 |
| IN 1-2 | IS 2 | 0.81928515 |
| IN 1-6 | IS 8 | 0.8191504 |
| IN 1-7 | IS 7 | 0.8191117 |
| IN 1-12 | IS 1 | 0.8169254 |
| IN 1-8 | IS 7 | 0.8153087 |
| IN 1-6 | IS 7 | 0.8146101 |
| IN 4-99 | IS 2 | 0.81453246 |
| IN 5-3 | IS 1 | 0.8143734 |
| IN 5-99 | IS 7 | 0.8142201 |
| IN 5-7 | IS 7 | 0.8139874 |
| IN 4-6 | IS 7 | 0.81257045 |
| IN 8-5 | IS 4 | 0.81216574 |
| IN 6-1 | IS 6 | 0.81212145 |
| IN 6-99 | IS 1 | 0.8117839 |
| IN 1-8 | IS 8 | 0.8116627 |
| IN 1-3 | IS 7 | 0.808799 |
| IN 8-99 | IS 4 | 0.8083526 |
| IN 4-8 | IS 1 | 0.8073891 |
| IN 1-9 | IS 8 | 0.8068104 |
| IN 1-4 | IS 8 | 0.8067572 |
| IN 99-14 | IS 2 | 0.806334 |
| IN 1-99 | IS 4 | 0.8057177 |
| IN 6-6 | IS 2 | 0.80404603 |
| IN 6-8 | IS 2 | 0.802497 |
| IN 8-13 | IS 4 | 0.8021523 |
| IN 8-11 | IS 7 | 0.8020085 |
| IN 8-8 | IS 4 | 0.80174536 |
| IN 8-8 | IS 2 | 0.8012606 |
| IN 6-4 | IS 5 | 0.80125093 |
| IN 6-7 | IS 8 | 0.8012167 |
| IN 4-99 | IS 8 | 0.8010602 |
| IN 5-5 | IS 7 | 0.8008286 |
| IN 1-11 | IS 8 | 0.8006029 |
| IN 4-7 | IS 7 | 0.79970217 |
| IN 6-9 | IS 4 | 0.7996293 |
| IN 4-8 | IS 7 | 0.799109 |
| IN 5-1 | IS 1 | 0.79796827 |
| IN 1-6 | IS 1 | 0.79774773 |
| IN 5-99 | IS 2 | 0.79732364 |
| IN 1-2 | IS 8 | 0.79663587 |
| IN 99-14 | IS 1 | 0.79641736 |
| IN 4-6 | IS 1 | 0.79536265 |
| IN 5-5 | IS 1 | 0.79532045 |
| IN 4-6 | IS 2 | 0.7947254 |
| IN 5-3 | IS 7 | 0.7937105 |
| IN 4-8 | IS 2 | 0.7930284 |
| IN 99-14 | IS 7 | 0.7927033 |
| IN 4-3 | IS 7 | 0.7920272 |
| IN 1-3 | IS 8 | 0.7918494 |
| IN 6-11 | IS 1 | 0.79140985 |
| IN 4-1 | IS 4 | 0.78997356 |
| IN 4-99 | IS 4 | 0.78814054 |
| IN 1-7 | IS 8 | 0.7865655 |
| IN 4-7 | IS 1 | 0.7863617 |
| IN 1-11 | IS 2 | 0.78588295 |
| IN 1-11 | IS 1 | 0.78575516 |
| IN 4-1 | IS 8 | 0.78547466 |
| IN 7-1 | IS 2 | 0.78516626 |
| IN 5-9 | IS 2 | 0.7839781 |
| IN 8-5 | IS 8 | 0.7835916 |
| IN 1-4 | IS 4 | 0.78295803 |
| IN 6-11 | IS 7 | 0.7825777 |
| IN 7-99 | IS 2 | 0.7820535 |
| IN 6-11 | IS 2 | 0.7818302 |
| IN 7-1 | IS 7 | 0.7816343 |
| IN 5-2 | IS 2 | 0.78097844 |
| IN 8-11 | IS 4 | 0.78004974 |
| IN 1-9 | IS 4 | 0.77993715 |
| IN 6-4 | IS 2 | 0.77990985 |
| IN 8-99 | IS 8 | 0.7795601 |
| IN 5-9 | IS 4 | 0.7790837 |
| IN 7-1 | IS 1 | 0.77855074 |
| IN 11-99 | IS 7 | 0.7784711 |
| IN 8-13 | IS 8 | 0.7782211 |
| IN 11-99 | IS 1 | 0.7775878 |
| IN 1-12 | IS 7 | 0.77662694 |
| IN 5-11 | IS 2 | 0.7762743 |
| IN 4-4 | IS 8 | 0.7762398 |
| IN 7-99 | IS 8 | 0.7755432 |
| IN 7-1 | IS 8 | 0.77534056 |
| IN 6-4 | IS 3 | 0.7752825 |
| IN 7-99 | IS 7 | 0.77517855 |
| IN 6-1 | IS 5 | 0.77481025 |
| IN 7-5 | IS 8 | 0.774704 |
| IN 4-6 | IS 8 | 0.77395606 |
| IN 7-1 | IS 4 | 0.7733123 |
| IN 6-10 | IS 8 | 0.77238804 |
| IN 8-5 | IS 2 | 0.7718595 |
| IN 8-11 | IS 1 | 0.7713287 |
| IN 6-7 | IS 7 | 0.77093023 |
| IN 8-10 | IS 4 | 0.77067673 |
| IN 1-7 | IS 4 | 0.77041095 |
| IN 5-99 | IS 8 | 0.76957184 |
| IN 6-10 | IS 4 | 0.76937246 |
| IN 5-7 | IS 1 | 0.76887244 |
| IN 8-99 | IS 2 | 0.76822186 |
| IN 4-5 | IS 2 | 0.7672707 |
| IN 5-3 | IS 2 | 0.766963 |
| IN 4-3 | IS 1 | 0.7669456 |
| IN 6-7 | IS 1 | 0.7665683 |
| IN 5-1 | IS 2 | 0.7662511 |
| IN 1-11 | IS 7 | 0.7637373 |
| IN 6-4 | IS 7 | 0.762867 |
| IN 11-99 | IS 8 | 0.7618176 |
| IN 4-7 | IS 4 | 0.76167136 |
| IN 99-14 | IS 8 | 0.7613318 |
| IN 1-2 | IS 7 | 0.7612256 |
| IN 4-8 | IS 8 | 0.76040053 |
| IN 5-9 | IS 8 | 0.7602143 |
| IN 6-4 | IS 1 | 0.759672 |
| IN 8-10 | IS 7 | 0.7593032 |
| IN 4-2 | IS 4 | 0.7585345 |
| IN 4-3 | IS 2 | 0.75845444 |
| IN 5-3 | IS 8 | 0.7583586 |
| IN 5-2 | IS 8 | 0.7572763 |
| IN 7-99 | IS 1 | 0.75719047 |
| IN 1-12 | IS 2 | 0.7571087 |
| IN 1-8 | IS 4 | 0.7570224 |
| IN 6-1 | IS 3 | 0.7564286 |
| IN 7-5 | IS 2 | 0.7560823 |
| IN 3-4 | IS 1 | 0.7555111 |
| IN 6-7 | IS 2 | 0.75516593 |
| IN 1-2 | IS 1 | 0.75510687 |
